# Protoplast Regeneration and Its Use in New Plant Breeding Technologies

**DOI:** 10.3389/fgeed.2021.734951

**Published:** 2021-09-03

**Authors:** Kelsey M. Reed, Bastiaan O. R. Bargmann

**Affiliations:** School of Plant and Environmental Sciences, College of Agriculture and Life Sciences, Virginia Tech, Blacksburg, VA, United States

**Keywords:** protoplast, regeneration, gene editing, crop improvement, tissue culture

## Abstract

The development of gene-editing technology holds tremendous potential for accelerating crop trait improvement to help us address the need to feed a growing global population. However, the delivery and access of gene-editing tools to the host genome and subsequent recovery of successfully edited plants form significant bottlenecks in the application of new plant breeding technologies. Moreover, the methods most suited to achieve a desired outcome vary substantially, depending on species' genotype and the targeted genetic changes. Hence, it is of importance to develop and improve multiple strategies for delivery and regeneration in order to be able to approach each application from various angles. The use of transient transformation and regeneration of plant protoplasts is one such strategy that carries unique advantages and challenges. Here, we will discuss the use of protoplast regeneration in the application of new plant breeding technologies and review pertinent literature on successful protoplast regeneration.

## Introduction

Since the advent of CRISPR/Cas9 and related gene-editing technology, direct modification of crop genomes has become the way of the future for advanced breeding techniques in agriculture (Zhang et al., 2019). These new plant breeding technologies (NPBT) have opened avenues of fundamental and translational research that were previously inaccessible. In contrast to transgenic approaches, NPBT can avoid costly and time-consuming regulatory hurdles and accelerate the introduction of new crop lines to the ag market (Lassoued et al., 2021).

**FIGURE 1:**
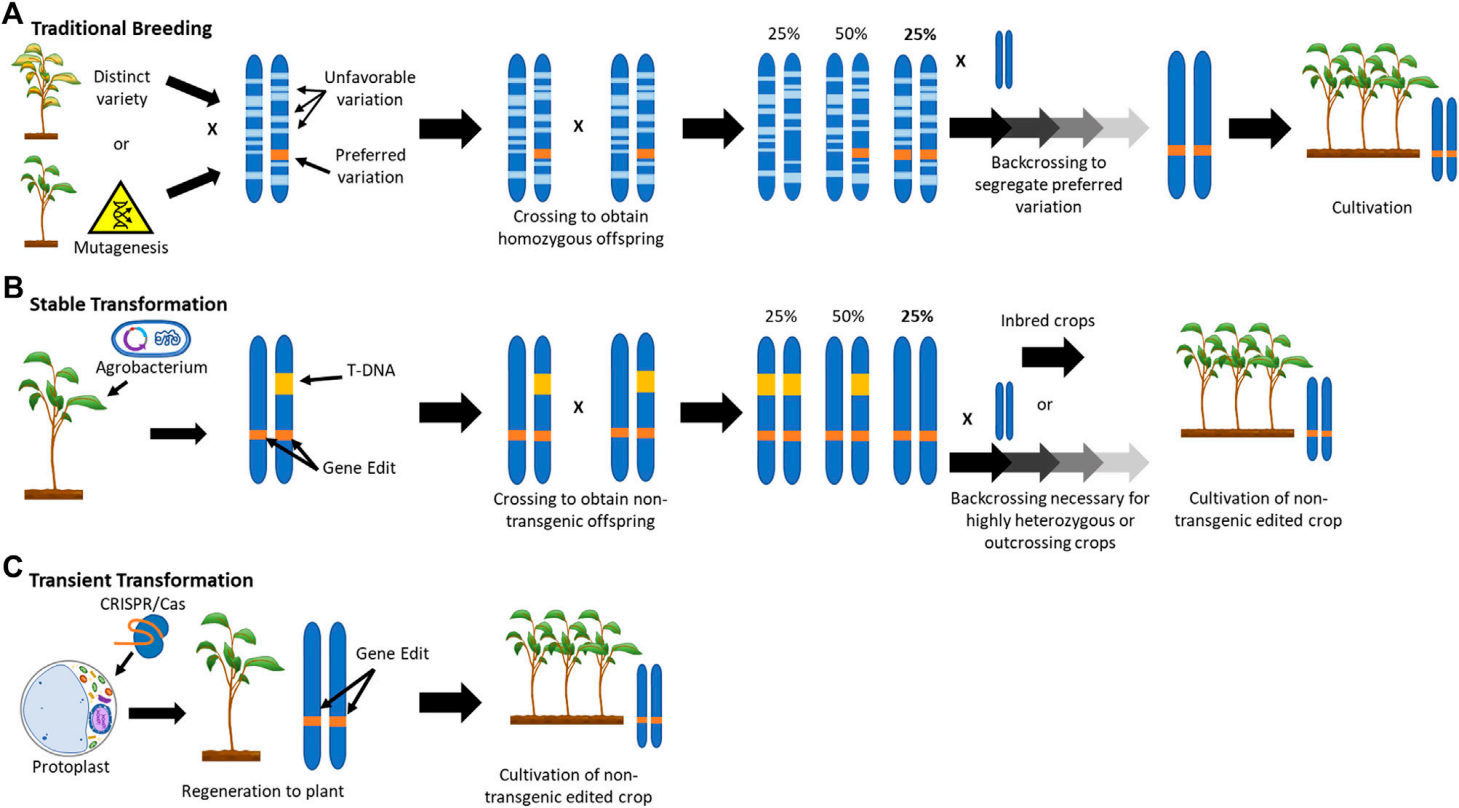
Schematic Representation of the Application of New Plant Breeding Technologies.

Breeding for the introgression of new traits from a wild relative or mutagenized population into an elite crop cultivar is a lengthy procedure, requiring numerous rounds of selection to regain the characteristics of the parental strain (Figure 1A). The ability to efficiently modify crop genes can save several years over conventional breeding approaches and phenotypic recurrent selection (Bull et al., 2017). However, the current most commonly used NPBT method of inserting a transgenic CRISPR/Cas9 construct into the host genome and then crossing it out again to obtain transgene-free progeny still requires multiple rounds of selection (Figure 1B). This is especially true for highly heterozygous and/or outcrossing crops.

In contrast to conventional breeding or transgenic CRISPR/Cas9 approaches, gene editing through transient transformation and regeneration of protoplasts can achieve the desired genetic outcome within a single clonal generation by avoiding the integration of foreign DNA into the host genome (Figure 1C). Aside from the potential to speed up the application of NPBT, the use of protoplasts may have numerous other advantages.

### Advantages of Using Protoplasts in NPBT

As stated above, the use of transient transformation of protoplasts can circumvent transgenesis (the integration of genetic material from one organism into the genome of another organism). The enzymatic removal of the plant cell wall allows for the introduction of foreign DNA, RNA, or protein into protoplasts through either polyethylene glycol (PEG) treatment or electroporation. Although relatively infrequent, the use of DNA (often in the form of plasmids) does not fully preclude the random integration of transgenes (Lin et al., 2018). However, CRISPR/Cas9 can also be expressed through transformation with mRNA encoding the Cas9 enzyme along with the desired guide RNA (gRNA) (Zhang et al., 2016). Alternatively, protoplasts can be transformed with ribonucleoprotein complexes, consisting of Cas9 associated with the gRNA (Svitashev et al., 2016). The latter two approaches more effectively preclude the integration of foreign DNA, although there have been cases where DNA-template contamination in the *in vitro* transcribed mRNA or gRNA has led to insertions, e.g. (Andersson et al., 2018). Particle bombardment is a potential alternative for transient delivery method for DNA-free gene-editing tools, e.g. (Liang et al., 2018). However, it may suffer from limitations in transformation efficiency and the regeneration of chimeric plants (as discussed below).

If the goal of the gene-editing approach goes beyond site-specific insertions and/or deletions for the knock-out of gene function but instead aims for specific nucleotide substitutions or insertion of a specific sequence through homologous recombination, there is a need for the co-introduction of a DNA-repair template (as in oligo directed mutagenesis) or a donor sequence, respectively. Prime editing and viral replicons are potential methods to deliver such templates and donors transgenically (Čermák et al., 2015; Lin et al., 2020). However, in addition to the potential for a non-transgenic outcome, the use of protoplasts allows for more control over the amount of template or donor delivered and effect higher precision and efficiency, e.g. (Sauer et al., 2016).

In many plant species, the lack of host susceptibility to *Agrobacterium* transformation limits the use of transgenic NPBT approaches. This is seen in particular in monocots (Hwang et al., 2017). Host-pathogen incompatibility is also expected to be a limiting factor in the applicability of viruses for the delivery of gene-editing tools (Ma et al., 2020). In such cases, the use of protoplasts (or particle bombardment) may be a feasible alternative delivery method.

Chimerism (where only parts of the regenerated plant are descended from an edited cell) can be an issue when using conventional, tissue-culture based approaches where a callus intermediate is used, e.g. (Charrier et al., 2019). This phenomenon occurs because *de novo* shoots or embryos can be formed from a group of cells rather than a single antecedent. In the case of protoplasts, regenerated plants are (in most cases) derived from a single cell, thereby avoiding this potential problem. Chimerism can be a concern especially when non-selectable, non-transgenic approaches are used together with conventional tissue culture, e.g. transient transformation with *Agrobacterium* or particle bombardment. Additionally, such non-selectable strategies can suffer from low editing efficiency in the regenerated plants because only the cells on the surface of the tissue are potentially edited whereas regeneration can also occur from the numerous non-transformed cells. In comparison, protoplast transformation efficiencies are much higher and plants regenerated from protoplasts transiently transformed with editing tools will therefore have better chance of being successfully edited.

However, a glaring limitation in the use of protoplasts for NPBT is the challenges faced in the regeneration of plants from single cells and there appears to be no universal strategy that applies to diverse (sub)species. Plant tissue culture in general, and protoplast regeneration in particular, is often lightheartedly considered more of an artform than a science, requiring an experienced eye and instinctual decision making, as comprehensive systematic approaches are too vast in scope to be feasible. In this review, we will discuss a compilation of literature on plant regeneration from protoplasts. We will deliberate protoplast isolation, protoplast culture, and plant regeneration from protoplast culture, specifically in the light of the application of NPBT.

## Obtaining Protoplasts

### Source Tissue

The tissue from which protoplasts are derived is very important for obtaining regenerable starting material. The genotype, organ or tissue, and growth conditions of the plants used can be a significant determinant in regeneration success.

#### Genotype

Different cultivars or ecotypes can have widely varying success rates in tissue culture and protoplast regenerative capacity. Depending on the species being worked with and the end goal of the application, it is recommended to assess the regenerative capacity of multiple genotypes and select the most suitable for further use.

When comparing four different Arabidopsis (*Arabidopsis thaliana*) ecotypes (Col-0, Ws-2, No-0, and HR-10), all gave a similar number of protoplasts with an optimized digestion, but differed significantly when comparing optimal protoplast division media, callus induction media, and shoot induction media (Jeong et al., 2021). Ws-2 showed the highest regeneration efficiency, whereas the Col-0, No-0, and HR-10 had relatively ineffective regeneration rates, regardless of efforts to vary the composition of media and tissue culture methods.

Comparison of three different *Cyclamen* species (*C. graecum*, *C. mirabile*, and *C. alpinum*) found significant differences in protoplast culture and regeneration, including division frequencies (often referred to as plating efficiency) and morphological appearance of regenerating embryos (Prange et al., 2010a). Plants were regenerated from protoplasts derived from embryogenic callus in all three species, but had different efficiencies in microcallus formation and development of somatic embryos. Interestingly, there was no correlation between the regenerative capacity of the source embryogenic callus and the ability of the protoplasts to divide and regenerate, with *C. graecum* performing the worst in regeneration from callus but showing the highest protoplast division rates.

#### Organ or Tissue

Different source materials for protoplast isolation can affect the number, size, viability, and regenerative capacity of protoplasts. There are examples of protoplast isolation and regeneration from numerous tissues, including leaves, cotyledons, roots, petioles, hypocotyls, petals, callus, and suspension cultures (Table 1).

**TABLE 1:** Obtaining Protoplasts.

Species	Tissue Source	Pre-digestion	Enzyme Composition	Digestion Buffer	Conditions	Yield	Reference
American Elm *(Ulmus americana)*	Cell suspension	None	0.2% cellulase Onozuka RS, 0.1% Driselase, 0.03% pectolyase Y-23	0.5 M mannitol, 2.5 mM MES, CPW salts	2 h, dark, 25°C	2 × 10^6^ per ml of packed cell volume	Jones et al. (2015)
Amur cork tree *(Phellodendron amurense)*	Callus	Sliced	1% cellulase Onozuka R-10, 1% Driselase	0.6 M mannitol	8 h	5.5 x 10^5^ gfw^−1^, 90% viability	Azad (2012)
Arabidopsis *(Arabidopsis thaliana)*	Seedlings	Plasmolysis	1% cellulase Celluclast 1.5 L, 2% carbohydrase Viscozyme L, 1% pectinase Pectinex ultra SP-L	0.47 M mannitol, 10 mM CaCl_2_, 10 mM MES	12 h, dark, 50 rpm, RT	1 × 10^7^ gfw^−1^	Jeong et al. (2021)
Banana *(Musa paradisiacal)*	Embryogenic cell suspension	None	3.5% cellulase R-10, 1% macerozyme R-10, 0.15% pectolyase Y-23	204 mM KCl, 67 mM CaCl_2_	10–12 h, dark, 50 rpm, 27°C	6 × 10^6^ per ml of packed cell volume	Dai et al. (2010)
Cabbage *(Brassica oleracea var. capitata)*	Cotelydons	Sliced and Plasmolysis	cellulase, pectinase (concentrations not disclosed)	0.5 M mannitol, 3 mM MES, CPW salts	Overnight, dark, 30 rpm, 25°C	not disclosed	Jie et al. (2011)
	Leaves	Sliced and Plasmolysis	0.5% cellulase Onozuka R-10, 0.1% pectolyase Y-23	0.4 M mannitol, 3 mM CaCl_2_, 2 mM MES	18 h, dark, 20 rpm, 25°C	not disclosed, 88% viability	Kiełkowska and Adamus (2019)
	Hypocotyls	Sliced and Plasmolysis	0.5% cellulase Onozuka R-10, 0.1% pectolyase Y-23	0.4 M mannitol, 3 mM CaCl_2_, 2 mM MES	18 h, dark, 20 rpm, 25°C	not disclosed, 92% viability	Kiełkowska and Adamus (2021)
	Leaves and hypocotyls	Sliced and Plasmolysis	1% cellulase Onozuka R-10, 0.1% macerozyme R-10	0.8 M sucrose, KM medium	16–18 h, dark, 30 rpm, 25°C	Leaves: 2 × 10^6^ gfw^−1^; Hypocotyls: 0.7 × 10^6^ fw^−1^; 60–90% viability	Kiełkowska and Adamus (2012)
Canola (*Brassica napus*)	Leaves	Sliced	1% cellulase Onozuka R-10, 0.1% macerozyme R-10	0.4 M sucrose, K3 medium	14–18 h, dark, 24°C	1 x 10^7^ gfw^−1^	Sahab et al. (2019)
Carrot *(Daucus spp.)*	Leaves	Sliced and Plasmolysis	1% cellulase Onozuka R-10, 0.1% pectolyase Y-23	0.6 M mannitol, 5 mM CaCl_2_, 10 mM MES	14–16 h, dark, 30 rpm, 26°C	not disclosed	Grzebelus and Skop (2014)
	Leaves and hypocotyls	Sliced and Plasmolysis	1% cellulase Onozuka R-10, 0.1% pectolyase Y-23	0.6 M mannitol, 5 mM CaCl_2_, 20 mM MES	14–18 h, 30 rpm, 26°C	Leaves: 3.21 x 10^6^ gfw^−1^, 74% viability; Hypocotyls: 0.96 x 10^6^ gfw^−1^	Grzebelus et al. (2012)
	Leaves	Sliced and Plasmolysis	1% cellulase Onozuka R-10, 0.1% pectolyase Y-23	0.6 M mannitol, 5 mM CaCl_2_, 20 mM MES	12–16 h, dark, 30 rpm, 26°C	2.8 x 10^6^ gfw^−1^, 72–93% viability	Maćkowska et al. (2014)
Cauliflower *(Brassica oleracea var. botrytis)*	Hypocotyls	Sliced and Plasmolysis	1% cellulase R-10, 0.1% macerozyme R-10	0.4 M sucrose, B5 salts and vitamins	15 h, dark, 24°C	5.2 x 10^6^ gfw^−1^	Sheng et al. (2011)
Chicory and Endive *(Cichorium intybus and endivia)*	Leaves	Sliced and Plasmolysis	1% cellulase Caylase 345, 0.5% pectinase Caylase M2	0.5 M mannitol, 30 mM sucrose, 0.55 mM inositol, 0.05 mM FeNa-EDTA, 1/2 MS macro elements, Heller micro elements, Morel & Wetmore vitamins	16 h, dark, 25 rpm, 23°C	1 x 10^6^ gfw^−1^, 85–95% viability	Deryckere et al. (2012)
Chrysanthemum *(Chrysanthemum morifolium)*	Leaves	Sliced and Plasmolysis	1.5% cellulase Onozuka R-10, 0.3% macerozyme R-10, 0.1% Driselase	0.4 M mannitol, 5 mM MES, CPW salts	4 h, dark, 40 rpm, 25°C	6.32 × 10^5^ gfw^−1^, 91.7% viability	Adedeji et al. (2020)
	Leaves and callus	Sliced and Plasmolysis	Leaves: 0.5% cellulase Onozuka R-10, 0.3% macerase R-10, 0.1% Driselase; Callus: 1.5% cellulase Onozuka R- 10, 0.5% macerase R-10, 0.1% Driselase	0.4 M mannitol	16 h, dark, 10 rpm, 22°C	not disclosed	Eeckhaut et al. (2020)
Coriander *(Coriandrum sativum vars.)*	Embryogenic cell suspension	None	2% cellulase Onozuka R-10, 1% pectinase and 0.2% macerozyme R-10	0.6 M mannitol, 5 mM CaCl_2_	14–18 h, dark, 50 rpm	4.81 × 10^6^ gfw^−1^, 90-93.8% viability	Ali et al. (2018)
Cottonwood *(Populus beijingensis)*	Cell suspension	None	1% cellulase Onozaka RS, 1% macerozyme R-10	0.6 M mannitol, 5 mM MES, CPW salts	4–6 h, dark, 80 rpm, 28°C	not disclosed, 90–95% viability	Cai and Kang (2014)
Crown imperial *(Fritillaria imperialis L.)*	Callus	Sliced	2% cellulase, 0.1% pectinase	0.5 M mannitol, CPW salts	8 h, dark, 70 rpm, 25°C	1.37×10^5^ gfw^−1^	Chamani and Tahami (2016)
Florist Kalanchoe *(Kalanchoe blossfeldiana)*	Cultured leaf explants	Sliced	0.4% cellulase Onozuka R-10, 0.2% Driselase	0.4 M mannitol, 100 mM glycine, 14 mM CaCl_2_, 0.5 mM MES, MS macro elements	4 h, dark, 40 rpm, 25°C	6.0 x 10^5^ gfw^−1^	Castelblanque et al. (2010)
Gentian *(Gentiana decumbens)*	Leaves	Plasmolysis	1% cellulase Onozuka R-10, 0.5% macerozyme R-10	0.5 M mannitol, 5 mM MES, CPW salts	3–4 h, dark, 50 rpm, 26°C	9.31 × 10^5^ gfw^−1^, 84.6% viability	Tomiczak et al. (2015)
Ginger *(Zingiber officinale Roscoe.)*	Embryogenic cell suspension	None	4.0% cellulase Onozuka R-10, 1.0% macerozyme R-10, 0.1% pectolyase	0.6 M mannitol, 0.45 M CaCl_2_, 5 mM MES	12–14 h, dark, 27°C	6.27 x 10^6^ gfw^−1^	Guan et al. (2010)
	Leaves and callus suspension	Sliced and Plasmolysis	1–3% cellulase Onozuka R-10, 0.5-1% macerozyme, 0-0.5% hemicellulase	0.5 M mannitol, CPW salts	10 h at 15°C followed by 6–8 h at 30°C, dark, 53 rpm	not disclosed	Nirmal Babu et al. (2016)
Grape hyacinth *(Muscari neglectum)*	Embryogenic callus	None	1% cellulase R-10, 1% Driselase, 0.1% pectolyase Y-23	0.5 M mannitol, 5 mM MES	2 h, dark, 90 rpm, 25°C	7 × 10^5^ gfw^−1^	Karamian and Ranjbar (2011)
Grapevine (*Vitis vinifera* L.)	Embryogenic callus	None	2% cellulase Onozuka, 1% macerozyme R-10, 0.05% pectolyase Y-23	0.5 M mannitol, 10 mM CaCl_2_, 5 mM MES	6 h, shaking	1 × 10^7^ gfw^−1^, >80% viability	Bertini et al. (2019)
Guava *(Psidium guajava)*	Leaves	Sliced	2.4% cellulase, 3% macerase, 0.6% hemicellulase	0.75 M mannitol, CPW salts	10 h, dark, 45 rpm, 27°C	3.7 x 10^6^ gfw^−1^, >90% viability	Rezazadeh and Niedz (2015)
Hydrangea *(Hydrangea spp.)*	Leaves	Sliced and Plasmolysis	0.002% cellulase Onozuka R-10, 0.0005% Driselase, 0.0005% MKC- hemicellulase, and 0.001% pectinase^a^	0.35 M sorbitol, 0.35 M mannitol, 9 mM CaCl_2_, 0.83 mM NaH_2_PO_4_, 3 mM MES	14–18 h, dark, 30 rpm, 25°C	5.5 × 10^6^ gfw^−1^, 87% viability	Kästner et al. (2017)
Lettuce (*Lactuca sativa*)	Cotelydons	None	1% cellulase R-10, 0.5% macerozyme R-10	0.45 M mannitol, 20 mM MES, CPW salts	14 h, dark, 40 rpm, 25°C	not disclosed	Woo et al. (2015)
	Leaves	Sliced	1.5% cellulase R-10, 0.3% macerozyme R-10	0.4 M mannitol, 20 mM KCl, 10 mM CaCl_2_, 20 mM MES, 0.1% BSA	4,5 h, dark, 50 rpm	not disclosed	Park et al. (2019)
Lily *(Lilium ledebourii)*	Leaves	Sliced	4% cellulase Onozuka R-10, 1% pectinase	0.7 M mannitol, CPW salts	24 h, dark, 70 rpm, 25°C	not disclosed	Tahami et al. (2014)
Love-in-a-Mist *(Nigella damascena L.)*	Callus	Sliced and Plasmolysis	1% cellulase, 0.1% pectolyase	0.6 M mannitol, 5 mM CaCl_2_, 10 mM MES	14–16 h, dark, 30 rpm, 26°C	3 × 10^5^ gfw^−1^	Klimek-Chodacka et al. (2020)
Oil palm *(Elaeis guineensis)*	Cell suspension	None	2% cellulase, 0.5% cellulase Onuzuka R10, 1% pectinase, 0.1% pectolyase Y23	0.2 M mannitol, 0.4 M KCl, 45 mM CaCl_2_	14 h, dark, 26°C	1.14 × 10^6^ gfw^−1^, 82% viability	Masani et al. (2013)
Petunia *(Petunia hybrids)*	Leaves	Sliced and Plasmolysis	2% cellulase Onozuka R-10 , 0.6% macerozyme R-10	0.6 M mannitol, 10 mM MES, 0.2% BSA	6 h, dark, 30 rpm, 25 °C	1.04 × 10^6^ gfw^−1^, 73.3% viability	Kang et al. (2020)
	Leaves	Sliced and Plasmolysis	1.2% carbohydrase Viscozyme, 0.6% cellulase Celluclast, 0.6% pectinase PectinEX	1 M Manitol, 8 mM CaCl_2_, 0.1 M MES, 0.1% BSA	3 h, 40 rpm, 25°C	6.9 × 106 per 12–16 leaves, 94.3% viability	Yu et al. (2020)
Qin-jiao *(Gentiana macrophylla)*	Embryogenic cell suspension	None	2% cellulase Onozuka R-10, 0.5 % macerozyme R-10, 0.5% hemicellulase	0.4 M sorbitol, 50 mM CaCl_2_, 2.5 mM MES	14–16 h, dark, 30 rpm, 25°C	6.2 x 10^6^ gfw^−1^, >90% viability	Hu et al. (2015)
Silk tree *(Albizia julibrissin)*	Leaves and callus	Sliced and Plasmolysis	Leaves: 1.5% cellulase Onozuka R-10, 1% pectolyase Y-23; Callus: 2% cellulase Onozuka R-10, 1% pectolyase Y-23	0.7 M mannitol, CPW salts	6 h (leaves) 16 h (callus), dark, 40 rpm, 25°C	Leaves: 6.31 x 10^5^ gfw^−1^, 87% viability; Callus: 5.53 x 10^5^ gfw^−1^, 85% viability	Rahmani et al. (2016)
Sowbread *(Cyclamen spp.)*	Somatic embryos and embryogenic cell suspension	Sliced and Plasmolysis for embryos only	2% cellulase R-10, 0.5% macerozyme R-10	0.35 M sucrose, KM8p macro elements	16–18 h, dark, 24°C	Suspension cultures: 4.24 x 10^5^ gfw^−1^; Somatic embryos: 0.57 x 10^5^ gfw^−1^; Dissected germinated embryos: 3.09 x 10^5^ gfw^−1^	Prange et al. (2010b)
	Embryogenic cell suspension	None	2% cellulase R-10, 0.5% macerozyme R-10	0.35 M sucrose, KM8p macro elements	16–18 h, dark, 24°C	1.36 × 10^6^ gfw^−1^	Prange et al. (2010a)
Stevia *(Stevia rebaudiana)*	Leaves	Sliced	2% cellulase Onozuka R-10, 1.5% macerozyme Onozuka R-10, 0.2% Driselase, 0.1% pectolyase Y-23	0.5 M mannitol, 2.5 mM CaCl_2_, 5 mM MES	4 h, dark, 55 rpm, 25°C	8.4 x 10^6^ gfw^−1^, 98.8% viability	Lopez-Arellano et al. (2014)
Strawberry *(Fragaria ananassa)*	Shoots	Sliced and Plasmolysis	1% cellulase Cellulysin, 0.1% pectinase Macerase	0.4 M sucrose, K3 medium	18 h, dark, 20 rpm, 25°C	not disclosed	Barceló et al. (2019)
Widow's-thrill *(Kalanchoë spp.)*	Leaves	Sliced and Plasmolysis	0.5% cellulase Onozuka R-10, 0.1% Driselase	0.58 M mannitol, 14 mM CaCl_2_, 93 mM glycine, 2.5 mM MES, 1.65 g/L MS macro elements	16–18 h, dark, 40 rpm, RT	10.78 × 10^5^ gfw^−1^, 60-90% viability	Cui et al. (2019)

a = suspicion of inaccurate magnitude reported.

In cabbage (*Brassica oleracea*), it was observed that hypocotyl-derived protoplasts yielded more regenerated shoots than leaf-derived protoplasts (Kiełkowska and Adamus, 2012). In a comparison on the regeneration capacity of protoplasts derived from leaves, cotyledons, and callus from coastal medick (*Medicago littoralis*), leaf protoplast-derived callus was found to have the highest regeneration capacity with a frequency of 20% and cotyledon protoplast-derived callus had a regeneration frequency of 15% (Zafar et al., 1995). In this study, callus-derived protoplasts developed only a few microcolonies that were not tested for regeneration. Embryogenic callus can potentially provide improved regeneration success in cases where somatic tissues fail to produce regenerable protoplasts, e.g*.* in grapevine (*Vitis vinifera*) (Bertini et al., 2019).

The age of the source tissue can also be of importance, both for protoplast yield and viability as well as regeneration success. Generally, protoplasts derived from younger tissues perform better in culture. This has been shown for hypocotyls and leaves in cabbage (Kiełkowska and Adamus, 2012) and cell suspension cultures in oil palm (*Elaeis guineensis*) (Masani et al., 2013), for example.

#### Plant Growth Conditions

The growth conditions of the starting material, including growth media and light, can have a significant effect on the regenerative capacity of protoplasts. An important consideration is that the material needs to be sterile (either grown under aseptic conditions or sterilized upon harvest) in order to be used for further culture of the obtained protoplasts.

In Arabidopsis, plants grown on Gamborg B5 medium and harvested 3 weeks after germination had a larger rosette with nearly twice as many leaves when compared to plant grown on Murashige and Skoog (MS) medium, resulting in twice as many protoplasts per harvested plant. However, during protoplast culture, the plants grown initially on MS media showed two to three times higher plating efficiency. And when comparing the photoperiod under which plants were grown, short day (10 h) resulted in a fourfold higher plating efficiency than long day (16 h) (Masson and Paszkowski, 1992).

Examination of cauliflower (*Brassica oleracea*) leaf protoplast quality of shoots grown in various vessel types found that protoplast yield, viability, division, and shoot regeneration was higher from tissue of plants grown in containers with vented lids compared to containers with closed lids (Chikkala et al., 2009).

### Enzymolysis

When it comes to isolating protoplasts, it is not only about obtaining a high number of protoplasts, but also about optimizing their viability and regenerative capacity. Many factors in the enzymolysis procedure may be of influence, including the utilized pretreatment, buffer composition, cell-wall digestion enzymes, incubation conditions, and purification methods (Table 1). Although, to our knowledge, there are not studies on the effect on protoplast regeneration directly for all of the different factors described here, it seems reasonable to assume that effects on the quality (viability) of the isolated protoplasts will translate to an influence on regenerative capacity of the isolated protoplasts.

#### Pretreatment

Pretreatment of tissue can be used to augment the number of viable protoplasts isolated by increasing the access of the used enzymes to the plant cell wall. This can be achieved through physical disruption of the tissue (e.g. slicing leaf tissue), vacuum infiltration of the enzyme solution, or a preplasmolysis treatment.

Slicing tissue into smaller sections or strips before moving to the enzyme solution allows for more surface area for the enzymes to work, leading to the release of more protoplasts. With rice (*Oryza sativa*), longitudinal cutting, parallel to the veins, before enzyme digestion resulted in over twice as many viable protoplast as leaves cut in cross section (Lin et al., 2018). Another example of physical disruption is the “Tape-Arabidopsis Sandwich” method (Wu et al., 2009). This method uses tape on both sides of a leaf to add support and allow the removal of the bottom epidermal layer. This protocol has been successfully applied to other Brassicaceae species, including *B. oleracea*, *B. napus*, *Cleome spinosa*, *C. monophilla*, and *C. gynadra* (Lin et al., 2018).

In addition to physical disruption, vacuum infiltration of plant tissue with the enzyme solution can be used to ensure that the enzymes are able to reach more of the cells, which could increase protoplast yield. In both apple (*Malus domestica*) and grapevine, vacuum infiltration was a part of the optimization of the protoplast isolation procedure to obtain the highest number of viable protoplasts per gram of fresh weight (Osakabe et al., 2018).

Preplasmolysis treatment is used to shrink the protoplasts away from the cell wall before introducing the enzyme solution. This is thought to avoid damage to the cell membrane. When comparing protoplasts isolated from birdsfoot trefoil (*Lotus corniculatus*) tissue with and without preplasmolysis, the pretreated protoplasts had roughly five times more cell wall formation than the nontreated after 3 days of culture. After 1 week, the viability of the nontreated protoplasts decreased significantly (Vessabutr and Grant, 1995).

#### Enzyme Solution Buffer

The buffer for the enzyme solution is critical for optimal enzyme activity and ensuring a high number of viable protoplasts. The buffer solution typically includes KCl; CaCl_2_; mannitol, sorbitol, or salts as osmolytes; MES (2-(N-morpholino)​ethanesulfonic acid) as pH buffer; BSA (bovine serum albumin) as an alternate target for proteases that may degrade the enzymes; and β-mercaptoethanol as a reducing agent (Table 1). Frearson et al. (1973) first formulated a combination of salts that many still use, called the cell and protoplast washing (CPW) salts. This basal salt solution is often modified with the addition of mannitol or sorbitol for osmotic pressure and different enzymes for optimal protoplast isolation (Jie et al., 2011; Jones et al., 2015; Tomiczak et al., 2015).

Proper osmolality is crucial in order to ensure the survival of the cells and provide an environment for potential cell wall formation and division, leading to regeneration. Protoplast development has been shown to be inhibited by excess osmotic pressure during isolation and culture by impairing metabolism (Ruesink, 1978) as well as division and cell wall regeneration (Pearce and Cocking, 1973).

Enzyme solutions with the same (or similar) composition as the subsequent protoplast culture medium have also been used successfully in protoplast regeneration applications. For example sugar beet (*Beta vulgaris*) callus protoplasts were isolated using Kao and Michayluk salts in the enzyme solution (Dovzhenko and Koop, 2003); Mango (*Mangifera indica*) pro-embryogenic mass-derived protoplasts were isolated using an enzyme solution containing Gamborg B5 and Murashige and Skoog salts (Ara et al., 2000); petunia (*Petunia* spp.) and calibrachoa (*Calibrachoa* spp.) leaf protoplasts were isolated with Kao and Michayluk and Gamborg B5 salts in the solution (Meyer et al., 2009).

#### Enzymes

Many commercially available cell-wall degrading enzymes (or enzyme mixtures) are used for the isolation of protoplasts. They differ in their substrates as well as the purity or combination of the enzymes in the extract. Enzymolysis is generally achieved using both cellulases and hemicellulases (e.g. beta-glucanases, xylanases, protopectinases, polygalacturonases, pectin lyases, and pectinesterases). Some of the most commonly used enzymes or enzyme mixtures are Cellulase R-10, Macerozyme R-10, and Pectolyase Y-23 (Table 1). The manufacturer/supplier of the enzymes may be a factor in the success rates (personal experience and communication with others).

The effect of different enzyme combinations and concentrations were tested on the isolation of protoplasts from stevia (*Stevia rebaudiana*) leaves (Lopez-Arellano et al., 2014). The optimized enzyme solution contained 2% Cellulase R-10, 1.5% Macerozyme Onozuka R-10, 0.2% Driselase, and 0.1% Pectolyase Y-23. When the Cellulase R-10 was decreased to 1% or increased to 3%, there was a significant drop in both the yield and viability of the protoplasts. There was also a lower viability when pectolyase Y-23 was not present. When isolating protoplasts from tobacco (*Nicotiana tabacum*) leaves, it was found that Pectolyase Y-23 was 20 times more effective than Macerozyme R-10 (Nagata and Ishii, 1979). This was determined to be due to the Pectolyase Y-23 having 50 times stronger endopolygalacturonase activity.

As the cost of lab-grade enzymes can be prohibitive, the use of food-grade cell wall degrading enzymes was investigated as a low-cost alternative for the isolation of switchgrass (*Panicum virgatum*) leaf protoplasts (Burris et al., 2016). It was determined that using a combination of Rohament CL with Rohapect 10 L and Rohapect UF (cellulases and pectinases commonly used in brewing and juicing) yielded up to 8.4 × 10^5^ protoplasts per gram of leaf tissue.

Although (to our knowledge) there have been no systematic analyses of whether the combination of enzymes used may influence the division rates and regenerative capacity of the produced protoplasts, one can imagine that there could well be an effect. The enzymes themselves, the crude extracts, as well as the cell-wall degradation products they produce can all be recognized by plant cells as pathogenic elicitors, to a greater or lesser extent, depending on the sensitivity of the genotype used to the different enzymes and extracts employed. Protoplast yield and viability may well be a good measure for protoplast isolation, but it could be the case that an enzyme combination that does not necessarily give the highest yield and viability could be more suitable for subsequent regeneration of the protoplasts.

#### Enzymolysis Conditions

Conditions during protoplast isolation (i.e. duration, temperature, light, and agitation) can play a significant role in the subsequent yield, viability and regenerative capacity of the protoplasts.

The length of a digestion period typically ranges from 2 to 18 h (Table 1). The duration of digestion needs to be long enough to release sufficient numbers of protoplasts, but not too long as to decrease the viability due to cell damage or the lack of nutrients and growth regulators in the enzymolysis solution. For example, when comparing 4, 8, and 12 h digestion duration of crown imperial (*Fritillaria imperialis*) callus, the yield and viability were highest at 8 h (Chamani and Tahami, 2016).

Temperature also plays an important role in protoplast yield and viability. Room temperature is the most commonly used, although there are examples of higher temperatures being employed (Table 1). There could be effects on enzyme activity (and protoplast yield) as well as protoplast viability and regenerative capacity. Intuitively, it may be preferable to use a temperature that is close to that used for the growth of the source material and/or subsequent protoplast culture conditions, in order to minimize temperature fluctuations or shocks. Conversely, perhaps a particular temperature treatment may actually benefit regenerative capacity.

Digestion in a light or dark condition may additionally influence the protoplast isolation, with most choosing dark conditions (Table 1). This may avoid the production of free radicals and photoinhibition in cells containing chloroplasts. Although there are also examples where digestion under light performed better than in the dark. In geranium (*Pelargonium* x *hortorum*) leaf protoplast isolation, protoplast yield and viability were increased when the digestion occurred in light; in the dark, the enzymes were efficient but most of the released protoplasts had burst (Nassour and Dorion, 2002). The protoplasts isolated from the light condition were regenerated into plants, but the effect of light or dark condition during digestion on the regeneration capacity was not investigated.

Agitation of the enzymatic solution on a gyratory shaker during the protoplast digestion can increase the protoplast yields. Typically, speeds range from 0 to 90 rpm, with the average being around 40 rpm (Table 1). Alternatively, the agitation can be implemented only at the end of the digestion period to facilitate the release of protoplasts from the cell wall remnants.

Again, protoplast yield and viability may well be a good measure, but it could be the case that digestion conditions that do not necessarily give the highest yield and viability could be more suitable for subsequent regeneration of the protoplasts.

#### Purification

Following enzymolysis, separation of the protoplasts from undigested tissue, cell wall debris, and dead cells can be an important factor in the culture of the protoplasts. Debris and dead cells may elicit negative effects in the living protoplasts that will inhibit their division and development, e.g. in kalanchoe (*Kalanchoe blossfeldiana*) (Castelblanque et al., 2010). Filtration and sucrose cushions, or floatation through a density gradient, are commonly used techniques.

## Protoplast Culture

### Culture Media

Protoplast culture media are central to protoplast division and plant regeneration. The appropriate macro-, micro-nutrients, and additives, such as plant growth regulators, osmotic stabilizers, medium solidifiers, and supplements, are essential in protoplast culture.

#### Nutrients

Optimal protoplast culture media vary widely, depending on the genotype and source tissue used (Table 2). Common medium formulations (such as MS (Murashige and Skoog, 1962), Gamborg (B5) (Gamborg et al., 1968), Kao and Michayluk (KM (Kao and Michayluk, 1975)), Y3 (Eeuwens, 1976), or Nitsch (Nitsch and Nitsch, 1969)), or slight modification thereof, are often used in protoplast culture. Although there are also examples of custom formulations, e.g. TM2G for tomato (*Solanum lycopersicon*) protoplast culture (Shahin, 1985). This is also a case where the manufacturer/supplier of the premixed media may be a factor in the success rates (personal experience and communication with others). When establishing and optimizing a protoplast culture procedure, it is prudent to assay an array of medium formulations for suitability.

**TABLE 2:** Protoplast Culture.

Species	Protoplast Density	Protoplast Culture Medium	Protoplast Culture PGRs	Time to Division	Time to Microcalli	Reference
American Elm *(Ulmus americana)*	2 × 10^5^ protoplasts/ml	Agarose beads (1.6% SeaPlaque agarose); liquid KM5/5 medium (KM medium, 10% mannitol, 2.56 mM MES)	5.4 μM NAA, 5 μM BAP	2-6 days	Not disclosed	Jones et al. (2015)
Amur cork tree *(Phellodendron amurense)*	4-6 x 10^5^ protoplasts/ml	Solid MS medium (3% sucrose, 0.2% Gellan gum)	4 μM NAA, 2 μM BAP	2 weeks	2 months	Azad (2012)
Arabidopsis *(Arabidopsis thaliana)*	1 x 10^6^ protoplasts/ml	Thin alginate layer (1.4% sodium alginate); liquid PIM medium (B5 medium, 2% sucrose, 6% myo‐inositol)	2.3 μM 2,4-D, 8.9 μM BAP	7 days	28 days	Jeong et al. (2021)
Banana *(Musa paradisiacal)*	1 × 10^6^ protoplasts/ml	Nurse culture: protoplasts in liquid M5 (MS medium, 4.5% sucrose, 4.1 μM biotin, 680 μM glutamine, 0.01% malt extraction) with a sterilized nitrocellulose filter seperating the feeder layer (MS medium, Morel vitamins, 4% sucrose, 0.25% myo-inositol, 9.05 μM 2,4-D, 2.8 mM glucose, 278 mM maltose, 1.2% agarose) containing the nurse cells (M. acuminate cv. Mas (AA))	4.5 μM 2,4-D	4-5 days	1 month	Dai et al. (2010)
Cabbage *(Brassica oleracea var. capitata)*	1-2 x 10^5^ protoplasts/ml	agarose embedding culture (MS medium (without NH_4_NO_3_), 8% myo-inositol, 3% sucrose, 1.19 mM thiamine), media with (20.6 mM ammonia (NH_4+_), 39.4 mM nitrate ions (NO_3-_)) added after 2 weeks	9.05 μM 2,4-D, 2.2 μM BAP	3-5 days	4 weeks	Jie et al. (2011)
	4 × 10^5^ protoplasts/ml	Alginate layers (1.4% alginic acid sodium salt); liquid culture medium (B5 medium, KM vitamins, 7.4% glucose, 0.025% casein hydrolysate, 0.1 μM PSK-α)	0.45 μM 2,4-D, 0.91 μM zeatin	3,4 days	3 weeks	Kiełkowska and Adamus (2019)
	4 × 10^5^ protoplasts/ml	Alginate layers (1.4% alginic acid sodium salt); liquid culture medium (B5 medium, KM vitamins, 7.4% glucose, 0.025% casein hydrolysate, 10 μM putrescine)	0.45 μM 2,4-D, 1 μM zeatin	3-5 days	4 weeks	Kiełkowska and Adamus (2021)
	4 × 10^5^ protoplasts/ml	Alginate layers (1.4% sodium alginate); CPP liquid medium (KM medium, MS FeEDTA, B5 vitamins, 7.4% glucose, 0.025% casamino acids)	0.45 μM 2,4-D, 0.91 μM zeatin	3,4 days	4 weeks	Kiełkowska and Adamus (2012)
Canola *(Brassica napus)*	5 x 10^5^ protoplasts/ml	Agarose (0.3% Sea-Plaque agarose) or Alginate (0.5% sodium alginate) Beads; liquid medium (combination of K3, H, and A mediums)	5.4 μM NAA, 0.45 μM 2,4-D, 0.89 μM BAP	6 days	3,4 weeks	Sahab et al. (2019)
Carrot *(Daucus spp.)*	4 x 10^5^ protoplasts/ml	Thin alginate layer; liquid CPP (KM medium, B5 vitamins, 7.4% glucose, 0.025% casein enzymatic hydrolysate, 100 nM PSK-α, 0.88 mM cefotaxime, 0.01-0.05% antibiotic (cefotaxime or timentin))	0.45 μM 2,4-D, 0.91 μM zeatin	5 days	Not disclosed	Grzebelus and Skop (2014)
	4 x 10^5^ protoplasts/ml	Calcium alginate layers (1.4% alginic acid sodium salt); CPP liquid medium (KM medium, B5 vitamins, 7.4% glucose, 0.025% casein hydrolysate)	0.45 μM 2,4-D, 0.91 μM zeatin	3 days	3-6 weeks	Grzebelus et al. (2012)
	4 x 10^5^ protoplasts/ml	Thin alginate layer; liquid CPP (KM medium, B5 vitamins, 7.4% glucose, 0.025% casein hydrolysate, 100 nM PSK-α, 0.88 mM cefotaxime, 0.3 mM timentin)	0.45 μM 2,4-D, 0.91 μM zeatin	4-8 days	Not disclosed	Maćkowska et al. (2014)
Cauliflower *(Brassica oleracea var. botrytis)*	2 x 10^5^ protoplasts/ml	Nurse culture: protoplasts in solid 1/2 medium (B5 medium, 4.5% sorbitol, 4.5% mannitol, 0.2% glucose, 0.2% agarose), suspended in liquid MS medium (7.3% mannitol) containing nurse cells (tuber mustard)	2.7 μM NAA, 4.5 μM 2,4-D, 2.2 μM BAP	3,4 days	21 days	Sheng et al. (2011)
Chicory and Endive *(Cichorium intybus and endivia)*	5 x 10^4^ protoplasts/ml	Low melting point agarose (LMPA) beads (0.25% LMPA); MC1 liquid medium (1/2 MS macro elements, Heller micro elements, Morel & Wetmore vitamins, 9% mannitol, 1% sucrose, 1.39 mM inositol, 2.55 mM glutamine, 0.05 mM FeNa-EDTA). After 5 days, MC1 liquid medium replaced with MC2 liquid medium (1/2 MS macro elements, Heller micro elements, Heller KCl, Morel & Wetmore vitamins, 6% mannitol, 1% sucrose, .55 mM inositol, 5.1 mM glutamine, 0.05 mM FeNa-EDTA)	MC1: 10.75 μM NAA, 4.45 μM BAP; MC2: 2.7 μM NAA, 2.2 μM BAP	Not disclosed	14 days	Deryckere et al. (2012)
Chrysanthemum *(Chrysanthemum morifolium)*	1 x 10^5^ protoplasts/ml	Liquid medium (1/2 MS medium (without NH_4_NO_3_), 7.2% mannitol, 1% sucrose, 5.13 mM MES, 0.02% activated charcoal)	10.75 μM NAA, 4.45 μM BAP	4,5 days	5 weeks	Adedeji et al. (2020)
	1 x 10^5^ protoplasts/ml	Liquid culture (1/2 MS salts (without NH_4_NO_3_), KM vitamins, 7.2% mannitol, 1% sucrose, 3.42 mM glutamine, 0.83 mM inositol, 5.13 mM MES)	10.75 μM NAA, 2.2 μM BAP	<1 week	5,6 weeks	Eeckhaut et al. (2020)
Coriander *(Coriandrum sativum vars.)*	2 × 10^5^ protoplasts/ml	Liquid MS medium	4.5 μM 2,4-D	Not disclosed	Not disclosed	Ali et al. (2018)
Cottonwood *(Populus beijingensis)*	2 × 10^5^ protoplasts/ml	Thin liquid culture (MS medium (without NH_4_NO_3_), 10.8% glucose)	9.05 μM 2,4-D, 0.89 μM BAP	4,5 days	5 weeks	Cai and Kang (2014)
Crown imperial *(Fritillaria imperialis L.)*	1 × 10^5^ protoplasts/ml	Liquid MS medium (MS medium, 9% mannitol, 0.02% casein hydrolysate)	2.3 μM 2,4-D, 4.45 μM BAP	48 h	3-4 weeks	Chamani and Tahami (2016)
Florist Kalanchoe *(Kalanchoe blossfeldiana)*	1 x 10^5^ protoplasts/ml	Liquid BMb medium (macronutrients (5 mM NH_4_NO_3_, 15 mM KNO_3_, 3 mM CaCl_2_, 1.5 mM MgSO_4_, 0.5 mM KH_2_PO_4_), MS micronutrients, SH vitamins (Shahin, 1985), 5.8% mannitol, 4.45% sucrose, 28 mM myo-inositol, 25 mM xylitol, 0.3 mM ascorbic acid, 0.05 mM adenine hemisulfate, 0.5 mM MES)	5.4 μM NAA, 2.3 μM 2,4-D, 2.2 μM BAP	5-7 days	30 days	Castelblanque et al. (2010)
Gentian *(Gentiana decumbens)*	1 × 10^5^ protoplasts/ml	Agarose beads (0.8% Sea Plaque Agarose); PCM liquid medium (MS medium (without NH_4_NO_3_), 3% glucose, 9% mannitol, 20.53 mM glutamine, 0.8% Sea Plaque Agarose)	10.75 μM NAA, 0.45 μM TDZ	3-5 days	10-12 weeks	Tomiczak et al. (2015)
Ginger *(Zingiber officinale Roscoe.)*	1 x 10^6^ protoplasts/ml	Shallow liquid MS medium (MS medium, 9% mannitol, 0.05% casein hydrolysate)	4.5 μM 2,4-D, 0.93 μM kinetin	2-4 days	10-12 weeks	Guan et al. (2010)
	Not disclosed	Liquid medium (MS medium, 7% mannitol, 2% sucrose)	2.7 μM NAA, 2.3 μM 2,4-D, 2.2 μM BAP	Not disclosed	Not disclosed	Nirmal Babu et al. (2016)
Grape hyacinth *(Muscari neglectum)*	1 x 10^5^ protoplasts/ml	Nurse culture: protoplasts were isolated in alginate beads (1% sodium alginate), suspended in liquid culture (MS medium, 9% mannitol, 0.57 mM ascorbic acid) with nurse cells (same species, 1 × 10^^6^ protoplasts/ml) also in alginate beads	5.4 μM NAA, 4.45 μM BAP	4-5 days	4-5 weeks	Karamian and Ranjbar (2011)
Grapevine *(Vitis vinifera L.)*	1 x 10^5^ protoplasts/ml	Disc-cultures: protoplasts in solid Nitsch’s medium (5.4% glucose, 3% sucrose, 0.2% gellan gum) suspended in liquid Nitsch’s medium (5.4% glucose, 3% sucrose, 0.3% activated charcoal)	10.75 μM NAA, 2.2 μM BAP	10 days	Not disclosed	Bertini et al. (2019)
Guava *(Psidium guajava)*	1 x 10^5^ protoplasts/ml	Alginate beads; liquid culture media (MS medium (without NH_4_NO_3_), 3% sucrose, 59.3 μM thiamine, 48.6 μM pyridoxine, 16.25 μM nicotinic acid, 22.8 μM pantothenic acid, 0.17 mM ascorbic acid, 10.25 μM glutamine, 0.56 mM myo-inositol, 0.43 mM proline)	5.4 μM NAA	Not disclosed	7 weeks	Rezazadeh and Niedz (2015)
Hydrangea *(Hydrangea spp.)*	1 x 10^5^ protoplasts/ml	Liquid PPM1 media (MS medium, MW vitamins, 0.5% sucrose, 9.5% mannitol, 0.5% PVP 10, 3.48 mM MES, 0.6 mM Timentin, 1.4 μM ascorbic acid, 0.13 mM citric acid, 67 nM Karrikinolide)	5.4 μM NAA, 2.2 μM BAP	3-15 days	3 weeks	Kästner et al. (2017)
Lettuce *(Lactuca sativa)*	2.5 x 10^5^ protoplasts/ml	Agarose layers (1.2% agarose); liquid medium (1/2 B5 medium, 10.3% sucrose, 3.38 mM CaCl_2_, 50 μM NaFe-EDTA, 1.67 mM sodium succinate, 0.51 mM MES)	0.9 μM 2,4-D, 1.33 μM BAP	Not disclosed	3 weeks	Woo et al. (2015)
				5 days	4 weeks	Park et al. (2019)
Lily *(Lilium ledebourii)*	1 × 10^6^ protoplasts/ml	Liquid medium (MS medium, 9% mannitol, 0.2% yeast extract)	4.5 μM 2,4-D, 0.93 μM kinetin	48 h	20 days	Tahami et al. (2014)
Love-in-a-Mist *(Nigella damascena L.)*	4 × 10^5^ protoplasts/ml	Alginate layers; CPP liquid medium (KM medium, B5 vitamins, 7.4% glucose, 0.025% casein hydrolysate)	5.4 μM NAA, 9.3 μM kinetin	10 days	Not disclosed	Klimek-Chodacka et al. (2020)
Oil palm *(Elaeis guineensis)*	5.7 x 10^5^ protoplasts/ml	Agarose bead culture (0.6% SeaPlaque agarose); Y3A liquid medium (2 μM GA_3_)	10 μM NAA, 2 μM 2,4-D, 10 μM IAA, 2 μM IBA, 10 μM Zea, 2 μM GA_3_ , 10 μM BA and 2 μM 2iP	9 days	Not disclosed	Masani et al. (2013)
Petunia *(Petunia hybrids)*	1 x 10^5^ protoplasts/ml	Liquid medium (KM medium, B5 vitamins, 10.9% mannitol, 1.0% sucrose, 5.13 mM MES)	5.4 μM NAA, 4.45 μM BAP	3 days	4 weeks	Kang et al. (2020)
	2.5 × 10^5^ protoplasts/ml	Liquid medium (MS medium, 6% myo-inositol, 2% sucrose)	9.05 μM 2,4-D, 2.2 μM BAP	1 day	3-4 weeks	Yu et al. (2020)
Qin-jiao *(Gentiana macrophylla)*	3–5 x 10^5^ protoplasts/ml	Agar-pool culture: protoplasts in liquid P1 (MS (without NH_4_NO_3_), 5.5% mannitol, 2% sucrose, 1% glucose, 20.53 mM glutamine, 0.05% casein hydrolysate) surronded by agar-solidified P1 (0.85% agar)	9.05 μM 2,4-D, 2.2 μM BAP	3-4 days	3-4 weeks	Hu et al. (2015)
Silk tree *(Albizia julibrissin)*	3-5 x 10^5^ protoplasts/ml	Agarose layers (1.4% SeaPlaque agarose); liquid KM8p medium (KM medium, 8% sucrose, 10.25 mM MES)	2.7 μM NAA, 2.2 μM BAP	30-48 h	4 weeks	Rahmani et al. (2016)
Sowbread *(Cyclamen spp.)*	1.5 x 10^5^ protoplasts/ml	Alginate films (1.15% sodium alginate); modified liquid KM8p medium (3.75 mM NH_4_NO_3_, 8.11 mM CaCl_2_)	2.3 μM 2,4-D, 1 μM 2iP	24-48 h	Not disclosed	Prange et al. (2010b)
	1.5 × 10^5^ protoplasts/ml	Species dependent: Agarose lense (1.5% LM agarose) or Alginate film (1.15% sodium alginate) in liquid medium, either 8 pmC.1 or 8 pmC.2 (modified KM8p, 3.75 mM NH_4_NO_3_, 8.11 mM CaCl_2_)	8 pmC.1: 4.5 μM 2,4-D, 0.4 2 μM 2iP; 8 pmC.2: 2.3 μM 2,4-D, 1 μM 2iP	24-48 h	Not disclosed	Prange et al. (2010a)
Stevia *(Stevia rebaudiana)*	5 x 10^5^ protoplasts/ml	Agarose bead culture (0.6% SeaPrep agarose); liquid modified KM8p medium (5.1% sucrose, 5.5% mannitol)	5.4 μM NAA, 0.9 μM 2,4-D, 2.28 μM zeatin	2,3 days	14 days to microcolonies	Lopez-Arellano et al. (2014)
Strawberry *(Fragaria ananassa)*	2 × 10^5^ protoplasts/ml	Agarose beads (0.6% agarose); modified KM8p liquid medium (7.2% glucose)	5.4 μM NAA, 1.14 μM TDZ	Not disclosed	3 weeks	Barceló et al. (2019)
Widow's-thrill *(Kalanchoë spp.)*	1 x 10^5^ protoplasts/ml	Liquid medium (KM medium, Schenk and Hildebrandt (1972) vitamins, 5% mannitol, 4% sucrose, 0.5% myo-inositol, 19.7 mM xylitol, 2.56 mM MES, 0.28 mM ascorbic acid, 27.1 μM adenine hemisulfate, 0.15 mM timentin)	5.4 μM NAA, 2.3 μM 2,4-D, 2.2 μM BAP	3-7 days	8 weeks	Cui et al. (2019)

In a comparison of 14 formulations based on MS, KM, and Y3 media for oil palm cell suspension-derived protoplast division, Y3-based medium gave the fastest cell wall formation, quickest division, and highest division frequency (Masani et al., 2013). Amur cork tree (*Phellodendron amurense*) stem protoplasts were cultured in MS, half-strength MS, and Woody Plant Medium (WPM), and culture in full-strength MS medium resulted in the highest colony formation rate (Azad, 2012).

Protoplast cultures also need a carbon source for energy metabolism, typically sucrose or glucose and to a lesser degree mannitol or sorbitol (Table 2). Comparing the effect of 1 and 2% of either glucose or sucrose as the carbon source for chrysanthemum (*Chrysanthemum morifolium*) leaf protoplast culture, 1% sucrose performed best (Adedeji et al., 2020). Although 2% sucrose resulted in the highest division rate, there was no subsequent colony formation. Only 1% sucrose and 2% glucose led to microcallus formation, with 1% sucrose more rapidly producing larger microcalli. For Arabidopsis seedling protoplast culture, three different variations of supplements with B5 medium and vitamins were tested for protoplast proliferation (Jeong et al., 2021). Myo-inositol as the primary carbon source along with sucrose resulted in the highest proliferation rate across four the different Arabidopsis ecotypes. A simplification of KM8p medium with the removal of all of the sugars (fructose, ribose, xylose, mannose, rhamnose, cellobiose, sorbitol and mannitol) except glucose still resulted in protoplast division that led to callus and embryo formation from carrot (*Daucus carota*) leaf protoplasts (Grzebelus et al., 2012).

#### Osmotic Pressure

Osmotic pressure is an important aspect of protoplast culture media. Generally, mannitol, sorbitol, sucrose, glucose, myo-inositol or a combination of these components is used to ensure the proper osmolarity. Determining the proper solute concentration is critical for the protoplast survival and division rates. Generally, the concentration of the major osmoticum used in the initial protoplast culture medium varies from 0.1 to 0.8 M (Table 2). Intuitively, it seems that having a comparable osmolarity between enzymolysis and initial culture conditions would expose the protoplasts to less osmotic shock upon transfer to culture medium and benefit their viability and vigor.

For cabbage cotyledon protoplasts, myo-inositol was a better osmotic regulator than mannitol (Jie et al., 2011). It is theorized that myo-inositol may be advantageous to both carbohydrate metabolism in cell walls and inositol metabolism in cell membranes in protoplast culture. However, whether these advantages are gained with a small addition of myo-inositol with a different primary osmoticum or if a large quantity of myo-inositol is needed has yet to be determined.

Osmolarity is commonly decreased gradually as the protoplast reform their cell walls and begin to divide. For example, gradually reducing the osmolarity for oil palm cell suspension protoplast cultures doubled the number of microcalli (Masani et al., 2013). In gentian (*Gentiana decumbens*) leaf protoplast culture, the osmolarity of the liquid medium around agarose beads was decreased by reducing the mannitol concentration from 0.5 to 0.33 M during the fifth and sixth week of culture, followed by another decrease to 0.17 M mannitol in the seventh and eighth week, and no mannitol for the subsequent weeks (Tomiczak et al., 2015). In chrysanthemum protoplast culture, after the first week in liquid culture medium, myo-inositol was omitted from the refresh medium and mannitol concentrations were dropped from the initial 0.4 M to 0.32, 0.21, and 0.11 M for weeks 2, 3, and 4, respectively (Eeckhaut et al., 2020).

#### Plant Growth Regulators

Plant growth regulators, particularly cytokinins and auxins, are essential for the growth of microcalli from protoplasts. Additionally, gibberellic acid (GA_3_) has been shown to be beneficial in some cases. The most common cytokinins are 6-benzylaminopurine (BAP), zeatin, kinetin, isopentenyl adenine (2iP), and thidiazuron (TDZ). The most common auxins are indole-3-acetic acid (IAA), indole-3-butyric acid (IBA), 2,4-dichlorophenoxyacetic acid (2,4-D), and naphthalene acetic acid (NAA). Optimal concentrations, combinations, and ratios vary widely, depending on the genotype and source tissue of the protoplasts (Table 2).

A ratio of a relatively higher concentration of auxin with a lower concentration of cytokinins was effective for microcallus formation from populus (*Populus beijingensis*) cell suspension protoplasts (Cai and Kang, 2014). Conversely, in kalanchoe leaf protoplast culture, a higher cytokinin to auxin ratio resulted in better proliferation and microcallus formation; having cytokinin exclusively resulted in slow growth and the microcalli eventually died (Castelblanque et al., 2010).

Coconut water is a natural source of plant growth regulators, both auxin (IAA) and cytokinins (various) as well as other phytohormones, such as gibberellins, and other supplements, such as vitamins and minerals, that have been found to be beneficial in plant tissue culture (Yong et al., 2009). As a supplement in corn (*Zea mays*) embryogenic callus protoplast culture, coconut water led to a high efficiency of microcallus formation, with a 2% coconut water addition producing the most microcalli (Imbrie-Milligan et al., 1987). Coconut water was also found to increase protoplast cell division in orchid (*Phalaenopsis* spp.) callus protoplasts (Kobayashi et al., 1993).

#### Additional Supplements

Additional supplements, such as polyvinylpyrrolidone, antioxidants, activated charcoal, silver nitrate, antibiotics, complex organics, amino acids, polyamines, conditioned medium, and peptide growth factors, can be added to the media to support protoplast division and microcallus formation (Table 2).

Antioxidants, such as ascorbic acid, citric acid, reduced glutathione, and L-cysteine, can be used to mitigate the inhibitory effects of reactive oxygen species. In oil palm protoplast regeneration, it was found that 200 mg/L ascorbic acid gave the greatest indication of further cell growth and development with the microcalli turning yellow and developing into embryogenic calli (Masani et al., 2013). With this supplementation, two types of embryogenic callus were observed, compact and friable embryogenic callus, which were both able to further develop into somatic embryos and regenerate into plantlets.

Polyvinylpyrrolidone (PVP) is used to adsorb phenolics. While phenolics may be beneficial for plant defense (Bhattacharya et al., 2010), an accumulation during protoplast culture has been found to lead to oxidative browning of the culture medium, inhibiting protoplast growth and division (Reustle and Natter, 1994; Prakash et al., 1997). There has also been reports of PVP suppressing tissue browning and improving callus formation in peony (*Paeonia lactiflora*) petal explant tissue culture (Cai et al., 2020). Polyvinylpolypyrrolidone (PVPP), a highly cross-linked version of PVP, has also been found to inhibit tissue necrosis in Virginia pine (*Pinus virginiana*) callus culture (Tang et al., 2004), as well as preventing browning better than PVP in guar (*Cyamopsis tetragonoloba*) cotyledon protoplast culture (Saxena and Gill, 1986). When PVP was added to the PVPP culture of guar cotyledon protoplasts, not only was it found to enhance the necrosis inhibition, but it also improved the protoplast division frequency. Another compound known to decrease tissue browning is 2-aminoindane-2-phosphonic acid (AIP), which is a reversible inhibitor of phenylalanine ammonia lyase (PAL), an enzyme necessary for polyphenol production (Appert et al., 2003). While the inhibition of PAL was able to increase the cell wall digestibility and facilitate sustained cell division in American elm (*Ulmus americana*), extended inhibition results in decreased shoot growth in tissue culture (Jones et al., 2012). This decrease in plant growth due to PAL inhibition from AIP has also been seen in birch (*Betula pubescens*) (Nybakken et al., 2007) and St. John’s wort (*Hypericum* spp.) (Klejdus et al., 2013). It could be hypothesized that an early addition of AIP will increase the likelihood of protoplast survival, but it should not be used for an extended period as to disrupt the callus and shoot growth, as described for American elm protoplast regeneration (Jones et al., 2015).

Activated charcoal is a commonly used additive employed for its ability to adsorb inhibitory elements, such as phenolics and reactive oxygen species, that can impede protoplast division. Adedeji et al. (2020) found that the ideal concentration of activated charcoal for chrysanthemum leaf protoplast regeneration was 0.02% (w/v) and adding a higher concentration of 0.1% resulted in agglutination of the protoplasts, causing them to die before entering the microcolony stage. In primrose (*Primula* spp.) cell suspension-derived protoplast culture, the addition of 0.1% PVP did not induce callus formation; however, the addition of activated charcoal did (Mizuhiro et al., 2001).

Silver nitrate (AgNO_3_), an inhibitor of ethylene action, has been shown in some cases to increase callus formation and regeneration efficiency as well as effect protoplast isolation efficiency. The culture of hypocotyl protoplasts from several *Brassica* species was markedly improved by the addition of silver nitrate in the culture medium (Pauk et al., 1991; Hu et al., 1999). With rice (*Oryza sativa*) suspension cultures, the addition of silver nitrate during protoplast isolation reduced protoplast yield but increased the frequency of colony formation (Ishii, 1988).

Antibiotics may be used to avoid endogenous or exogenous contamination, however they can either inhibit or stimulate explant growth and development with the direct causation not yet understood (Qin et al., 2011). A study analyzing the effects of three β-lactam antibiotics (cefotaxime, carbenicillin, and timentin) at different concentrations on carrot seedling protoplasts found that, while plating efficiencies decreased in all antibiotic concentrations higher than 100 mg/L, cefotaxime and timentin in the range of 100–500 mg/L increased regeneration efficiency (Grzebelus and Skop, 2014). Timentin was used with *Hydrangea* leaf protoplasts to limit the endophytes and it was observed that in antibiotic-free medium, the protoplasts rebuilt the cell wall faster and divided earlier, but callus was only formed in medium with antibiotics (Kästner et al., 2017).

The exact composition of complex organics, such as casein hydrolysate, casamino acids, coconut water, and yeast extract, is typically undefined and varies depending on the manufacturer/supplier and potentially the batch. However, the amino acids, hormones, vitamins, fatty acids, carbohydrates, and other growth supplements they provide may enhance growth and regeneration of plants (Bhatia, 2015). The addition of casein hydrolysate was initially shown to give a more consistent high rate of microcallus formation from tobacco (*Nicotiana tabacum*) protoplasts (Galun and Raveh, 1975), and is currently an addition to protoplast culture media regularly (Table 2).

Polyamines can regulate plant growth and stress responses through many means, including increasing antioxidant activity and regulating oxidative stresses (Chen et al., 2019). In a comparison of the exogenous addition of the polyamines putrescine, spermidine, and spermine on sugar beet (*Beta vulgaris*) cell suspension-derived protoplasts, spermine resulted in the highest plating efficiency, likely due to its stronger inhibitory effect on ethylene production (Majewska-Sawka et al., 1997). Polyamines exogenously applied in different concentrations on cabbage hypocotyl protoplast culture obtained the highest frequency of shoot organogenesis from protoplasts treated with putrescine (Kiełkowska and Adamus, 2021). However, the addition of putrescine had no effect on the culture or regeneration of Love-in-a-Mist (*Nigella damascena*) callus protoplasts (Klimek-Chodacka et al., 2020).

Conditioned medium (spent liquid medium used for cell-suspension cultures that is filtered and subsequently used as a supplement for protoplast culture) may contain compounds that encourage growth and mitotic activity. Fresh conditioned medium from cell-suspension cultures significantly increased the plating efficiency in chrysanthemum leaf protoplast culture (Zhou et al., 2005).

Phytosulfokine (PSK), specifically PSK-α, is a peptide that was originally detected secreted in conditioned medium, but was later found in whole plants (Yang et al., 1999). It was found to promote cell growth, enhance callus growth as well as adventitious root and bud formation, and improve somatic embryogenesis in multiple species, and has also been shown to enhance protoplast regeneration in carrot (Maćkowska et al., 2014) and cabbage (Kiełkowska and Adamus, 2019). With carrot leaf protoplasts, application of PSK-α during the initial culture resulted in a four-fold increase in regenerated plants (Maćkowska et al., 2014). PSK-α was shown to be both genotype- and dose-dependent and did not require a constant presence to maintain cell divisions in cabbage leaf protoplasts (Kiełkowska and Adamus, 2019). Not only was the PSK-α found to promote cell proliferation, but it also increased differentiation and organogenesis in five of the six cabbage accessions tested.

### Protoplast Culture Conditions

Protoplast culture conditions, such as the use of liquid or semi-solid medium, temperature and light, cell density, or the presence of nurse cultures, can have a significant effect on the division and microcallus formation potential of protoplasts.

#### Liquid Vs. Semi-solid Medium

When it comes to determining the solidity of the media to use with protoplast culturing, there are multiple factors to consider, including imaging potential, media refreshing, toxin accumulation, and cell aggregation.

Liquid medium is the most straightforward to make since it requires no agar manipulation. However, it faces a multitude of challenges. With imaging, unless each cell is in a separate space, it is impossible to track the growth of an individual cell. There is also the potential for aggregation of cells to form a non-homogeneous callus, possibly resulting in chimerism of the regenerated plants. Aggregation can also cause a local accumulation of toxic substances released from dying cells that may inhibit the growth of neighboring cells (Deryckere et al., 2012).

To avoid cell agglutination, embedding the protoplasts in semi-solid medium can ensure physical separation of cells. The embedding medium will typically contain agar, agarose, or alginate as a solidifier. Alginate is favorable for heat-sensitive protoplasts because the gelling is induced by exposure to calcium ions rather than the need to heat the agar or agarose solutions above the melting point.

In a comparison between thin alginate layers and extra thin alginate films on carrot shoot protoplast culture, thin alginate layers resulted in nearly a 20% increase in plating efficiency in every accession tested (Maćkowska et al., 2014). Sterilizing the alginate solution through filter-sterilization was also found to give over a 10% increase in plating efficiency over autoclave-sterilization in several of the accessions used.

The amount of liquid medium surrounding alginate beads can affect the protoplast proliferation capability. In American elm (*Ulmus americana*) cell suspension-derived protoplast alginate bead culture, cultures that contained less than 2 ml or more than 3 ml of liquid medium failed to develop beyond the first cell division; whereas cultures that contained 2 or 3 ml of liquid medium continued to proliferate (Jones et al., 2015).

#### Temperature and Light

The temperature and light conditions used during protoplast culture vary widely (Table 2) and have both been shown to be of effect in regeneration success. Cabbage leaf protoplast cultures were greatly affected by light and temperature, with very few divisions occurring in cultures moved from dark at 25°C to light at 23°C after 7 days of culture, compared to those kept in the dark conditions for all 15 days (Kaur et al., 2006). Using lettuce (*Lactuca saligna*) leaf protoplasts, dark culture led to sustained division while light bleached and killed the protoplasts in 3 days (Brown et al., 1987). However, Arabidopsis cotyledon protoplasts did not show a significant variation in either the plating density or growth rates whether cultured in the light or dark (Dovzhenko et al., 2003).

#### Cell Density

The protoplast plating density can range from single cells up to a few million protoplasts per milliliter, but typically range from 5 × 10^4^–1 × 10^6^ protoplasts/ml (Table 2). In a comparison of plating densities of petunia (*Petunia hybrida*) leaf protoplast culture, 1 × 10^6^ protoplasts/ml produced a significantly higher division frequency and number of calli than 5 × 10^4^ protoplasts/ml (Kang et al., 2020). However, the microcolony viability decreased with the plating density increasing to 1.5 × 10^6^ protoplasts/ml, potentially due to high phenolics accumulation. Over-crowding the protoplasts can also result in a lower viability due to a lack of available nutrients (Kiełkowska and Adamus, 2012). In contrast, a lower density may also be desired to track an individual protoplast after transformation or fusion (Bhojwani and Dantu, 2013). However, a lower protoplast density can be more costly and time consuming. Additionally, protoplasts can release growth factors which can stimulate mitotic division non-cell-autonomously. This is also the basis for nurse cultures.

#### Nurse Cultures

Nurse cultures are the culture of target protoplasts with additional actively dividing protoplasts or suspension cells, either from the same species (e.g. in crocus (*Crocus cancellatus*) embryogenic calli-derived protoplast culture (Karamian and Ebrahimzadeh, 2001)) or from another, often closely related species (e.g. in desert banana (*Musa paradisiacal*) embryonic cell suspension protoplast culture (Dai et al., 2010) and cauliflower (*Brassica oleracea* var. *botrytis*) hypocotyl protoplast culture (Sheng et al., 2011)). There are many nurse culture techniques, one example is feeder layer-cultures, which can be embedding the target protoplasts in agar layers with the nurse cells in a liquid surrounding the layers (Sheng et al., 2011), or the target protoplasts in liquid culture with the nurse cells embedded in agarose (Dai et al., 2010). Alginate bead cultures, which can be performed by embedding the target protoplasts in alginate beads and having the nurse cells in liquid medium (e.g*.* with rice (*Oryza sativa*) suspension culture protoplasts (Kyozuka et al., 1987)). An alternate method for ensuring a separation of the nurse cells and the target protoplasts is using a nitrocellulose filter which allows growth factors, signaling molecules, and nutrients to pass through, but not cells (Dai et al., 2010).

### Plant Regeneration From Protoplast Culture

#### Callus Formation

From microcalli, regeneration could come from organogenesis or embryogenesis. Organogenesis-oriented microcalli can be moved to a callus proliferation medium to increase the callus size, whereas embryogenesis-oriented microcalli can be moved to embryo formation medium; however, either could also proliferate callus or form embryos on the microcallus medium, depending on the genotype, source tissue, and medium composition.

Organogenesis typically relies on moving callus to a medium containing both a cytokinin and auxin or a shooting medium followed by a rooting medium. When it comes to the timeframe for regeneration, it is difficult to directly compare organogenesis and embryogenesis between different species and source tissues (Table 3). Intuitively, embryogenesis should take less time than organogenesis due to the extended time the callus needs to shoot and then root versus an embryo’s ability to grow and differentiate both organs at the same time.

**TABLE 3:** Regeneration from Protoplast Culture.

Species	Callus Proliferation/Embryo Formation Medium	Callus/Embryo PGRs	Time to Calli/Embryo	Regeneration Medium	Regeneration PGRs	Time to Regeneration	Regeneration Process	Reference
American Elm *(Ulmus americana)*	Microcalli not transferred	N/A	Not disclosed	Shoots: solid ESM medium (DKW medium (Driver and Kuniyuki, 1984), 3% sucrose, 0.3 μM GA_3_, 0.22% Phytagel); Roots: solid RM medium (DKW medium (Driver and Kuniyuki, 1984), 3% sucrose, 0.6% activated charcoal, 0.22% Phytagel)	Shoots: 2.2 μM BAP; Roots: 0.5 μM IBA	4–6 weeks from calli to shoots; 1,2 months from shoots to roots	Organogenesis	Jones et al. (2015)
Amur cork tree *(Phellodendron amurense)*	Microcalli not transferred	N/A	4 months to calli	Solid MS medium (MS medium, 3% sucrose, 0.2% Gellan gum)	Shoots: 2 μM BAP and 1 μM NAA or 2.5 μM IBA; Roots: 2 μM IBA	5 weeks from callus to shoots; 1 week from shoots to roots	Organogenesis	Azad (2012)
Arabidopsis *(Arabidopsis thaliana)*	Callus induction medium (B5 medium, 2% sucrose)	2.3 μM 2,4-D, 8.9 μM BAP	2,3 weeks from microcalli to calli	Shoots: Shoot induction medium (MS medium, 3% sucrose, 2.41 mM MES, 0.8% plant agar); Roots: rooting medium (1/2 MS medium containing vitamin, 1% sucrose, 2.41 mM MES, 0.8% plant agar)	Shoots: 0.9 μM IAA, 2.5 μM 2iP; Roots: 5 μM IBA	0–3 weeks from transfering calli to shoots; 2 weeks from shoots to roots	Organogenesis	Jeong et al. (2021)
Banana *(Musa paradisiacal)*	Solid M6 (MS medium, 3% sucrose, 0.2% gelrite)	2.3 μM IAA, 2.2 μM BAP	3 months to germinated embryos	Solid rooting media (MS medium, 0.1% activated charcoal, 3% sucrose, 0.7% agar)	None	1 month from germinated embryo to plantlet	Embryogenesis	Dai et al. (2010)
Cabbage *(Brassica oleracea var. capitata)*	Solid MS medium (MS medium, 3% sucrose, 8% myo-inositol, 0.4% Gelrite)	9.05 μM 2,4-D, 2.2 μM BAP	Not disclosed	Shoots: MS medium; Roots: half-strength MS medium	Shoots: 2.7 μM NAA, 8.9 μM BAP and ; Roots: none	3 weeks from calli to shoots	Organogenesis	Jie et al. (2011)
	Microcalli not transferred	N/A	Not disclosed	MS medium (0.1 μM PSK-α)	None	4-6 weeks from calli to shoots	Organogenesis	Kiełkowska and Adamus (2019)
	Microcalli not transferred	N/A	4-6 weeks from microcalli to calli	Shoots: Solid MS2 medium (MS medium, 2% sucrose, 0.25% Gelrite); Roots: MS medium	Shoots: 2.7 μM NAA, 8.8 μM BAP; Roots: none	Not disclosed	Organogenesis	Kiełkowska and Adamus (2021)
	Microcalli not transferred	N/A	Not disclosed	Solid MS medium (MS medium, 2% sucrose, 0.25% Phytagel)	None	4 weeks from calli to shoots	Organogenesis	Kiełkowska and Adamus (2012)
Canola *(Brassica napus)*	Microcalli proliferation medium (MS medium, 3.5% sucrose, 2.56 mM MES, 0.7% agarose)	5 μM NAA, 5 μM 2,4-D, 5 μM BAP	1 week from microcalli to calli	Shoots: shoot regeneration medium (SRM) (MS medium, 3% sucrose, 2.56 mM MES, 0.05% PVP, 29.4 μM silver nitrate, 0.3 μM GA_3_, 0.7% agarose); shoot elongation medium (SEM) (MS medium, B5 vitamins, 2% sucrose, 2.56 mM MES, 0.1 μM GA_3_, 0.8% agar); Roots: root induction media (RIM) (1⁄2 strength MS, B5 vitamins, 1% sucrose, 2.56 mM MES, 0.6% agar)	SRM: 0.5 μM NAA, 2.5 μM 2iP; SEM: 2 μM BAP; RIM: 2.5 μM IBA	4-6 weeks from calli to shoots; 4 weeks for shoot elongation; 3-7 days from elongated shoots to roots	Organogenesis	Sahab et al. (2019)
Carrot *(Daucus spp.)*	Microcalli not transferred	N/A	2 months to calli and embryos	Solid R medium (MS medium, 2% sucrose, 0.3 mM thiamine, 0.49 μM pyridoxine, 4.06 μM nicotinic acid, 40 μM glycine, 0.56 mM myo-inozytol, 0.25% phytagel)	None	2,3 weeks from calli/embryos to plantlets	Undetermined	Grzebelus and Skop (2014)
	Microcalli not transferred	N/A	Not disclosed		None	Somatic embryos; 1 month from calli to plants; 2-3 months total to plantlet	Embryogenesis	Grzebelus et al. (2012)
	Microcalli not transferred	N/A	2 months to calli and embryos		None	5 weeks from calli or embryo to plantlet	Undetermined	Maćkowska et al. (2014)
Cauliflower *(Brassica oleracea var. botrytis)*	Microcalli not transferred	N/A	5–7 weeks to calli	Solid regeneration medium (MS medium, 3% sucrose, 0.8% plant agar)	4.6 μM zeatin and 1.15 μM IAA	10 weeks to shoots	Organogenesis	Sheng et al. (2011)
Chicory and Endive *(Cichorium intybus and endivia)*	Microcalli not transferred	N/A	Not disclosed	Solid MC3 medium (1/2 MS macro elements, Heller micro elements, Morel & Wetmore vitamins, 1% sucrose, .55 mM inositol, 0.05 mM FeNa-EDTA, 0.5% agar)	2.85 μM IAA, 2.2 μM BAP	14 weeks total to plantlet	Organogenesis	Deryckere et al. (2012)
Chrysanthemum *(Chrysanthemum morifolium)*	Soild proliferation medium (1⁄2 MS medium, 2% sucrose, 0.25% gelrite)	10.75 μM NAA, 4.45 μM BAP	Not disclosed	Shoot induction medium (MS medium, 3% sucrose, 0.3% gelrite)	Shoots: 2.7 μM NAA, 4.45 μM BAP; Roots: 9.05 μM 2,4-D, 13.3 μM BAP	16 weeks from calli to plantlet	Organogenesis	Adedeji et al. (2020)
	Semi-solid proliferation media (1/2 MS salts, KM vitamins, 1% sucrose, 26.6 μM glycine, 0.4% Phytagel)	0.11 μM NAA, 2.2 μM BAP	2 weeks from microcalli to calli	Regeneration media (MS medium, KM vitamins, 2% sucrose, 26.6 μM glycine, 0.6% MC29 agar)	0.45 μM TDZ	Not disclosed	Organogenesis	Eeckhaut et al. (2020)
Coriander *(Coriandrum sativum vars.)*	Microcalli not transferred	N/A	3,4 weeks to calli; 4 weeks from calli to embryos	MS medium (1.44 μM GA_3_)	4.45 μM BAP	4,5 months to outdoor plant	Embryogenesis	Ali et al. (2018)
Cottonwood *(Populus beijingensis)*	Callus proliferation media (MS medium (without NH4NO3), 3% sucrose, 0.6% agar)	4.52 μM 2,4-D, 0.89 μM BAP	Not disclosed	Shoots: MS medium; Roots: rooting medium (1/2 MS medium, 3% sucrose, 0.6% agar)	Shoots: 2.22 μM BA, 0.54 μM NAA; Roots: 2.46 μM IBA	4 weeks from calli to shoots; 12 weeks totals to shoots	Organogenesis	Cai and Kang (2014)
Crown imperial *(Fritillaria imperialis L.)*	Microcalli not transferred	N/A	Not disclosed	Solid MS medium	2.7 μM NAA, 6.66 μM BAP	Not disclosed	Organogenesis	Chamani and Tahami (2016)
Florist Kalanchoe *(Kalanchoe blossfeldiana)*	Colonies cultured in liquid BMb for 15 days, then added liquid BMc (MS medium, SH vitamins (Shahin, 1985), 3.8% mannitol, 3% sucrose, 0.6 mM myo-inositol) for calli proliferation, then small calli moved to solid BMc (0.8% agar)	5.4 uM NAA and 8.9 uM BAP	Not disclosed	Solid BMa (MS medium, ST vitamins (Staba, 1969), 3% sucrose, 0.6 mM myo-inositol, 3 μM thiamine, 0.8% agar)	0.6 μM IAA	5 months total to plantlet	Organogenesis	Castelblanque et al. (2010)
Gentian *(Gentiana decumbens)*	Agar-solidified CPM3 (MS medium, 3% sucrose, 0.217 mM adenine sulfate), then non-embryo calli moved to agar-solidified PRM3 (MS medium, 3% sucrose, 2% coconut water, 1.44 μM GA_3_, 0.217 mM adenine sulfate) for embryo formation	CPM3: 0.54 μM NAA, 8.9 μM BAP, 4.53 μM dicamba; PRM3: 4.65 μM kinetin	Somatic embryos 6 weeks on CPM3 or 12 weeks on CPM3/PRM3	Agar-solidified half-strength MS medium (1/2 MS medium, 1.5% sucrose)	None	Not disclosed	Embryogenesis	Tomiczak et al. (2015)
Ginger *(Zingiber officinale Roscoe.)*	Solid MS medium (MS medium, 3% sucrose, 0.7% agar)	0.9 μM 2,4-D, 22.2 μM BAP	6 months to embryos	MS medium	Shoots: none; Roots: 3.22 μM NAA, 8.9 μM BAP	15 months total to plantlet	Embryogenesis	Guan et al. (2010)
	Microcalli not transferred	N/A	40–60 days to calli	Solid MS medium (MS medium, 4% mannitol, 3% sucrose)	5.4 μM NAA, 4.45 μM BAP	Not disclosed	Organogenesis	Nirmal Babu et al. (2016)
Grape hyacinth *(Muscari neglectum)*	Solid half-strength MS agar medium	Callus proliferation: 0.45 μM BAP; Embryo formation: none	Not disclosed	half strength MS medium	4.45 μM BAP	3 months from embryo to plantlet	Embryogenesis	Karamian and Ranjbar (2011)
Grapevine (*Vitis vinifera* L.)	Embryo germination medium (Nitsch’s medium, 3% sucrose, 0.2% gellan gum)	None	3-4 months to embryos; 4 weeks for embyo germination	Shoots: C2D4B medium (C2D medium, 3% sucrose, 0.7% TC agar); Roots: MS medium (3% sucrose, 0.7% TC agar)	Shoots: 4 μM BAP; Roots: 0.5 μM NAA	3-4 weeks from germinated embryo to shoots; 6 month total to outdoor plants	Embryogenesis	Bertini et al. (2019)
Guava *(Psidium guajava)*	Solidified culture media (8% agar)	5.4 μM NAA	Not disclosed	Shoots: shoot regeneration medium; Roots: MS medium (medium specifics not disclosed)	Shoots: 11.15 μM kinetin, 7.1 μM BAP; Roots: 0.5 μM IBA	8 weeks from microcalli to shoots; 4 weeks from shoots to roots	Organogenesis	Rezazadeh and Niedz (2015)
Hydrangea *(Hydrangea spp.)*	Solid PPM3 medium (MS medium, MW vitamins, 0.5% PVP 10, 3.48 mM MES, 3% sucrose, 5% mannitol, 0.6 mM Timentin, 1.4 μM ascorbic acid, 0.13 mM citric acid, 67 nM Karrikinolide, 0.25% Phytagel)	10.75 μM NAA with 8.9 μM BAP	8 weeks from microcalli to calli	Solid SRM medium (B5 salts and vitamins, 2.18% sucrose, 0.615 mM myo-inositol, 0.6 mM Timentin, 0.142 mM ascorbic acid, 0.13 mM citric acid, 0.068% Gelrite, 0.3% bactoagar)	0.54 μM NAA, 8.9 μM BAP	15 months total to plantlet	Organogenesis	Kästner et al. (2017)
Lettuce (*Lactuca sativa*)	Microcalli not transferred	N/A	Not disclosed	Shoots: Regeneration medium (MS medium, 3% sucrose, 0.6% plant agar); Roots: 1/2 MS medium	Shoots: 0.54 μM NAA, 2.2 μM BAP; Roots: none	4 weeks from microcalli to shoots	Organogenesis	Woo et al. (2015)
	Microcalli not transferred	N/A	Not disclosed	Shoot induction medium (MS medium, 3% sucrose, 0.6% agar); Roots: MS medium	Shoots: 0.54 μM NAA, 2.2 μM BAP; Roots: none	4 weeks from calli to shoots	Organogenesis	Park et al. (2019)
Lily *(Lilium ledebourii)*	Microcalli not transferred	N/A	Not disclosed	semi-solidified MS medium	0.54 μM NAA, 6.66 μM BA	Not disclosed	Organogenesis	Tahami et al. (2014)
Love-in-a-Mist *(Nigella damascena L.)*	Embryo formation media (MS medium, 3% sucrose, 0.7% agar)	5.4 μM NAA, 9.3 μM kinetin	3 months to calli; 3 weeks from calli to embryo	Regeneration media (MS medium, 13.4 μM glycine, 2% sucrose, 0.2% phytagel)	None	2 months from embryo to plantlet	Embryogenesis	Klimek-Chodacka et al. (2020)
Oil palm *(Elaeis guineensis)*	Solid Y3 medium (1.14 mM ascorbic acid)	1 μM NAA, 0.1 μM BAP	4-12 weeks to calli; 20-24 weeks from calli to embryos	ECI solid medium (media specifics not disclosed)	1 μM NAA and 0.1 μM BAP	12 weeks from embryo to plantlet; 56-68 weeks total to plantlet	Embryogenesis	Masani et al. (2013)
Petunia *(Petunia hybrids)*	KM proliferation medium (KM medium, B5 vitamin, 3.0% sucrose)	2.7 μM NAA, 2.2 μM BAP	4 weeks from microcalli to calli	MS medium (MS medium, 3% sucrose, 0.8% plant agar)	Shoots: 1 μM IBA, 4.45 μM BAP; Roots: none	Not disclosed	Organogenesis	Kang et al. (2020)
	Callus induction medium (MS medium, 3% sucrose)	2.7 μM NAA, 8.9 μM BAP	2,3 weeks from microcalli to calli	Regeneration medium (MS medium, 3% sucrose)	Shoots: 4.6 μM zeatin; Roots: none	2,3 weeks from calli to shoots	Organogenesis	Yu et al. (2020)
Qin-jiao *(Gentiana macrophylla)*	Solidified MS medium (MS medium, 3% sucrose, 0.05% casein hydrolysate, 0.85% agar)	Callus proliferation: 9.05 μM 2,4-D, 2.2 μM BAP; Embryo formation: 2.3 μM 2,4-D	6 weeks from microcalli to proembryos	Solidified MS medium (MS medium, 0.05% casein hydrolysate, 3 % sucrose, and 0.85 % agar)	Germination: 8.9 μM BAP; Rooting: none	2 weeks from proembryo to germination; 3 weeks from germination to plantlet	Embryogenesis	Hu et al. (2015)
Silk tree *(Albizia julibrissin)*	Solid MSB5 medium (MS medium, B5 vitamins, 3% sucrose, 0.02% casein hydrolysate)	10.8 μM NAA, 4.4 μM BAP	Not disclosed	Shoots: MS medium; Roots: half-strength MS medium	Shoots: 4.6 μM zeatin, 13.2 μM BAP; Roots: 4.9 μM IBA	5 weeks from calli to shoots; 4,5 weeks from shoots to roots	Organogenesis	Rahmani et al. (2016)
Sowbread *(Cyclamen spp.)*	Callus proliferation: 2.31.S medium (1/2 MS medium, 0.38% Gelrite); Embryo formation: 2.25.S medium (2× MgSO4 , 2× CaCl_2_, 2× microelements, 0.3% Gelrite) (medium specifics not disclosed)	2.31.S: 4.5 μM 2,4-D, 2 μM 2iP; 2.25.S: none	8-16 weeks to calli; 8-16 weeks from calli to embryos	2.41.S (3× CaCl_2_, 0.1% activated charcoal) (medium specifics not disclosed)	None	2.5 weeks from germinated embryo to plantlet	Embryogenesis	Prange et al. (2010a)
	Solid 2.1.S (1/2 MS medium, 0.38% Gelrite) (medium specifics not disclosed)	9.05 μM 2,4-D, 4 μM 2iP	Not disclosed	Solid 2.2.S (half-strength MS medium, 2 x CaCl_2_, 0.37% Gelrite) (media specifics not disclosed)	None	16 weeks from calli to plantlet; 24-28 weeks total to plantlet	Embryogenesis	Prange et al. (2010b)
Stevia *(Stevia rebaudiana)*	Solidified MS medium (MS medium, 3% sucrose, 0.05% casein hydrolysate, 0.3% Gelrite)	0.45 μM 2,4-D, 4.45 μM BAP	4 weeks to embryogenic calli	MS medium	5.4 μM NAA	8 weeks from embryogenic calli to plantlet; 1 month from embryo to plantlet	Embryogenesis	Lopez-Arellano et al. (2014)
Strawberry *(Fragaria ananassa)*	Agar-solidified modified KM8p medium (KM medium, 6.8% sucrose)	0.54 μM NAA, 4.45 μM BAP	Not disclosed	Solid MS medium (MS medium, 6.8% sucrose, 0.3% agarose)	1.08 μM NAA, 13.62 μM TDZ	4 weeks from calli to shoots; 16 weeks total to shoots	Organogenesis	Barceló et al. (2019)
Widow's-thrill *(Kalanchoë spp.)*	Liquid media (MS medium, 3% sucrose, 0.56 mM myo-inositol, 2.56 mM MES, 3.5% mannitol, 0.15 mM timentin)	5.4 μM NAA, 2.3 μM 2,4-D, 2.2 μM BAP	Not disclosed	Shoots: solidified MS medium (MS medium, 3% sucrose, 3.5% mannitol, 0.56 mM myo-inositol, 2.56 mM MES, 0.15 mM timentin, 0.3% gelrite); Roots: solidified MS medium (MS medium, 3% sucrose, 2.56 mM MES, 0.15 mM timentin, 0.3% gelrite, 0.1% Atamon)	Shoots: 5.4 μM NAA, 8.9 μM BAP or 9.12 μM zeatin; Roots: IAA	17-21 weeks total to shoots	Organogenesis	Cui et al. (2019)

Embryogenic callus formation can be from somatic protoplasts (somatic embryogenesis) or from embryogenic callus-derived protoplasts (secondary embryogenesis). Embryogenesis relies on cells within the microcalli presenting embryogenic properties, i.e. isodiametric, cytoplasm-rich cells (Dai et al., 2010). The embryogenic microcalli can then proliferate into embryogenic callus or form embryos directly. Embryos that form from the (micro)callus can be moved to media for germination and plantlet maturation.

#### Rooting and Shooting Media

When it comes to regenerating plants from protoplast-derived callus, either embryogenic or somatic callus, the media composition can determine the efficiency of the regeneration. A majority of methods use solid MS media supplemented with auxin and cytokinin (Table 3). Typically, shooting is the primary goal with rooting coming shortly after, then planting in soil for maturation. It is generally easier to get roots from shoots than shoots from roots.

In cabbage leaf protoplast shoot regeneration, MS versus Gamborg B5 based media supplemented with PSK-α and with or without plant growth regulators was compared (Kiełkowska and Adamus, 2019). Microcolonies were freed from alginate layers and, after transferring to regeneration medium, the callus would turn green, remain white, or begin to brown. The browning callus was considered dead, the white callus grew slightly but did not form shoots, and the green callus led to shoot regeneration roughly 4–6 weeks after transfer. It was found that the highest shoot regeneration came from callus placed on MS media with PSK-α and without PGRs across a majority of the genotypes tested.

When determining the effect of cytokinin on shoot induction from guava (*Psidium guajava*) leaf protoplast-derived callus, BAP and kinetin concentrations were investigated (Rezazadeh and Niedz, 2015). Concentrations of 7.1 μM BAP and 11.15 μM kinetin were optimal for shoot production; a higher concentration did not significantly increase the number of shoots. It was also found that changing the kinetin level was more effective than BAP.

Some methods involve the addition of other supplements to the regeneration medium to assist the callus growth and differentiation. Activated charcoal is a common addition, with its ability to prevent browning of callus by adsorbing growth inhibitors (Prange et al., 2010a; Masani et al., 2013). Masani et al. (2013) also examined the effects of ascorbic acid to reduce discoloration and promote embryogenesis. They found that ascorbic acid increased the number of embryogenic calli which subsequentially improved the regeneration efficiency of oil palm embryogenic cell suspension-derived protoplasts.

#### Somaclonal Variation

Somaclonal variation is the genetic or phenotypic variation that occurs in plants from tissue culture. A phenotypic change can be explained by either a genetic or epigenetic modification. Somaclonal variation can influence the fertility of the regenerant as well as the potential for changing the ploidy level, which is crucial for breeding.

Somaclonal variation is a potential occurrence in protoplast regeneration that can reveal itself in morphological or ploidy variation (Prange et al., 2010b; Sheng et al., 2011; Grzebelus et al., 2012; Tomiczak et al., 2015; Barceló et al., 2019). In strawberry (*Fragaria ananassa*), morphological differences between the control and regenerated protoclones were observed (including plant size and leaflets per leaf) that were not explained by ploidy level changes but rather genetic variation detected by microsatellite markers (Barceló et al., 2019). Prange et al. (2010b) and Tomiczak et al. (2015) both collected regenerated plants that were tetraploid from protoplasts that were initially diploid. In *Cyclamen coum*, it was observed that a single callus would give rise to both tetraploid and diploid regenerants which was reasoned could be a result from either the chromosomes doubling during callus culture or an error in separation of callus during culturing (Prange et al., 2010b). With *Gentiana decumbens*, there was no morphological difference in the regenerants, besides wider leaf blades (Tomiczak et al., 2015), yet 100% of the regenerated plants were tetraploid.

When considering the culture method’s role in this somaclonal variation, one hypothesis is that if genome duplication occurred during protoplast culture, it is most likely due to the possibility that tetraploid protoplasts divide faster than diploid protoplasts, as shown in tobacco (*Nicotiana plumbaginifolia*) (Magnien et al., 1982) and rapeseed (*Brassica napus*) (Magnien et al., 1982; Chen et al., 1994). If the polyploidization occurred during callus formation, the hypothesis is endoreduplication (amplification of DNA without mitosis) in callus cells, shown previously in pea (*Pisum sativum*) (Ochatt et al., 2000) and barrelclover (*Medicago truncatula*) (Elmaghrabi and Ochatt, 2006) and would explain this increase of DNA content. It has also been shown that plant growth regulators typically added to protoplast culture media have an effect on endoreduplication frequency in sugar beet (*Beta vulgaris*) (Lukaszewska et al., 2012).

Time in tissue culture increases chances of somaclonal variation. Isolating protoplast from plant tissue may therefore be favorable over isolating from callus tissue in order to avoid somaclonal variation due to the additional *in vitro* step that is required to obtain callus. This additional step has the potential to introduce genetic variation and effect the protoplast regeneration efficiency.

While somaclonal variation is undesirable in commercial crop production, it does have the benefit of creating phenotypic variability with a large number of regenerants that can be obtained through protoplast regeneration. This gives the potential for the identification of mutations that could be beneficial for a variety of uses, such as biotic resistance (Grzebelus et al., 2013), abiotic resistance (Kiełkowska et al., 2019), or create a desirable ornamental property.

## Protoplast Transformation

Electroporation as a method for protoplast transformation is not as popular as PEG-mediated transformation. With electroporation, there are more factors to consider that potentially have effects on transfection efficiency and cell survival: pulse voltage, pulse length, pulse number, cell number, DNA concentration, and electroporation buffer composition (Lee et al., 2020). However, when optimized, electroporation can be very efficient. Lee et al. (2020) found that when electroporation transformation was optimized for cabbage protoplasts, the transformation efficiency was nearly double that of PEG-mediated delivery, although both transformation rates were low (3.4 and 1.8%, respectively). Wójcik and Rybczyński (2015) studied the effect of electroporation the culture of embryogenic cell suspension-derived protoplasts from gentian (*Gentiana kurroo*). A high electric field voltage over 1 kV/cm significantly decreased protoplast survival and division. A single pulse had nine-fold higher protoplast viability than two pulses. Comparing the effect of length of the electric pulse on protoplast viability, it was found that 5 ms completely killed the protoplasts and 40 μs was too long and resulted in no division of the protoplast. A 20 μs pulse had the highest protoplast viability and division, 70 and 44.5% respectively. Significantly higher protoplast viability was obtained with an electroporation buffer with KCl, higher MgCl_2_ and pH, and lower MES (Wójcik and Rybczyński, 2015).

The more common PEG-mediated transformation requires less materials than electroporation but does require chemicals that could potentially damage the protoplasts. The main factors to consider with regards to transformation efficiency and cell survival are PEG concentration, transfection time, DNA concentration, and cell number (which has previously been shown to influence the results (Burris et al., 2016)). Transformation with PEG can reach a high transformation rate, such as 90% in petunia leaf protoplasts (Subburaj et al., 2016) and 80% in both wheat leaf protoplasts and rice sheath protoplasts (Shan et al., 2013). Although, a high transformation rate does not translate to a large number of transformed regenerants. For example, petunia leaf protoplasts transiently transformed with PEG for CRISPR/Cas9 ribonuclear protein multiplexing of two genes had a 55% transfection efficiency, but only eight of the 67 regenerated plants (11.9%) had indel mutations (Yu et al., 2020). PEG-mediated transformation of potato (*Solanum tuberosum*) leaf protoplasts resulted in more callus formation when treated with 12.5% PEG than 20% PEG; however, even the 12.5% PEG treatment resulted in a ten-fold decrease in callus formation compared to the untreated control (Craig et al., 2005).

## Outlooks and Obstacles

In our opinion, the use of protoplast regeneration in NPBT has a promising future. It has been used in numerous applications of gene-editing for crop trait improvement; e.g. the knock-out of the BRASSINOSTEROID INSENSITIVE 2 (BIN2) gene in lettuce (*Lactuca sativa*) (Woo et al., 2015) and the granule bound starch synthase (GBSS) gene in potato (Andersson et al., 2018) or the oligo-directed mutagenesis of the 5-enolpyruvylshikimate-3-phosphate synthase (EPSPS) gene in flax (*Linum usitatissimum*) (Sauer et al., 2016). We expect to see many more examples of its successful application in the coming years.

Nonetheless, there are obstacles that need to be addressed in order to overcome some of the challenges associated with protoplast regeneration. It is a process that demands specialized tissue culture expertise, requires complex manipulation, and can be time-consuming. Overall, current methods for protoplast regeneration are very genotype-specific and need to be made more universal for increased applicability and success.

One potential approach for making protoplast regeneration universally available is to gain fundamental knowledge of the transcriptional regulation of the regeneration process via transcriptomic analysis. While transcriptomic analysis of protoplast culture (e.g. in moss (*Physcomitrella patens*) protonema protoplasts for the initial 72 h of culture (Xiao et al., 2012)) has previously been investigated, there is a lack of and difficulty in knowing the transcriptional activity of solely protoplasts destined for regeneration. Single-cell transcriptome profiling has been demonstrated (Shulse et al., 2019), but the question remains on how to differentiate between protoplasts with regeneration capability and the larger, doomed protoplast population. Additional challenges arise when taking the cell-type composition of the source organ as well as the genotype into account.

Another process that can potentially improve universal application of protoplast regeneration technologies is through ectopic expression of embryogenic or morphogenic factors. Theoretically, if an ample number of protoplasts can directly develop into embryos, the regeneration frequency would multiply, resulting in a large number of regenerated plantlets. The direct development of protoplasts into embryos could also decrease the time in tissue culture, reducing the potential of somaclonal variation. The embryogenic or morphogenic transcription factors would need be to be transiently expressed in order to avoid any developmental effects that constitutive expression may cause (e.g*.* ectopic expression of BABY BOOM causing embryogenic growth on vegetative tissue (Boutilier et al., 2002)). Identification of appropriate embryogenic or morphogenic transcription factors, which could function individually or as a collective, as well as the timing of expression would need to be investigated. Recently, a study using Arabidopsis mesophyll protoplasts showed that timed transcriptional activation of auxin biosynthesis can significantly enhance regeneration success (Sakamoto et al., 2021). It will be interesting to see whether this approach is applicable to divergent species.

## References

[B1] Adedeji O. S. Naing A. H. Kim C. K. (2020). Protoplast Isolation and Shoot Regeneration from Protoplast-Derived Calli of Chrysanthemum Cv. White ND. Plant Cel. Tiss. Organ. Cult 141, 571–581. 10.1007/s11240-020-01816-3

[B111] Ali M. Mujib A. Zafar N. Tonk D. (2018). Protoplast Isolation and Plant Regeneration in Two Cultivated Coriander Varieties, Co-1 and RS. bta 99, 345–355. 10.5114/bta.2018.79965

[B2] Andersson M. Turesson H. Olsson N. Fält A.-S. Ohlsson P. Gonzalez M. N. (2018). Genome Editing in Potato via CRISPR-Cas9 Ribonucleoprotein Delivery. Physiol. Plantarum 164, 378–384. 10.1111/ppl.12731 29572864

[B3] Appert C. Zoń J. Amrhein N. (2003). Kinetic Analysis of the Inhibition of Phenylalanine Ammonia-Lyase by 2-Aminoindan-2-Phosphonic Acid and Other Phenylalanine Analogues. Phytochemistry 62, 415–422. 10.1016/s0031-9422(02)00561-7 12620354

[B4] Ara H. Jaiswal U. Jaiswal V. S. (2000). Plant Regeneration from Protoplasts of Mango ( Mangifera Indica L.) through Somatic Embryogenesis. Plant Cel. Rep. 19, 622–627. 10.1007/s002990050783 30754827

[B5] Azad M. A. K. (2012). Plant Regeneration through Callus-Derived Protoplasts of Phellodendron Amurense Rupr. BioTechnology 6, 317–326.

[B6] Barceló M. Wallin A. Medina J. J. Gil-Ariza D. J. López-Casado G. Juarez J. (2019). Isolation and Culture of Strawberry Protoplasts and Field Evaluation of Regenerated Plants. Scientia Horticulturae 256, 108552. 10.1016/j.scienta.2019.108552

[B7] Bertini E. Tornielli G. B. Pezzotti M. Zenoni S. (2019). Regeneration of Plants from Embryogenic Callus-Derived Protoplasts of Garganega and Sangiovese grapevine (Vitis vinifera L.) Cultivars. Plant Cel. Tiss. Organ. Cult. 138, 239–246. 10.1007/s11240-019-01619-1

[B8] Bhatia S. (2015). “Plant Tissue Culture,” in Modern Applications of Plant Biotechnology in Pharmaceutical Sciences. Editors Bhatia S. Sharma K. Dahiya R. Bera T. (Boston: Academic Press), 31–107. 10.1016/b978-0-12-802221-4.00002-9

[B9] Bhattacharya A. Sood P. Citovsky V. (2010). The Roles of Plant Phenolics in Defence and Communication during Agrobacterium and Rhizobium Infection. Mol. Plant Pathol. 11, 705–719. 10.1111/j.1364-3703.2010.00625.x 20696007 PMC6640454

[B10] Bhojwani S. S. Dantu P. K. (2013). “Parasexual Hybridization,” in Plant Tissue Culture: An Introductory Text. Editors Bhojwani S. S. Dantu P. K. (India: Springer), 173–198. 10.1007/978-81-322-1026-9_14

[B11] Boutilier K. Offringa R. Sharma V. K. Kieft H. Ouellet T. Zhang L. (2002). Ectopic Expression of BABY BOOM Triggers a Conversion from Vegetative to Embryonic Growth. Plant Cell 14, 1737–1749. 10.1105/tpc.001941 12172019 PMC151462

[B12] Brown C. Lucas J. A. Power J. B. (1987). Plant Regeneration from Protoplasts of a Wild Lettuce Species (Lactuca Saligna L.). Plant Cel. Rep. 6, 180–182. 10.1007/bf00268472 24248645

[B13] Bull S. E. Alder A. Barsan C. Kohler M. Hennig L. Gruissem W. (2017). FLOWERING LOCUS T Triggers Early and Fertile Flowering in Glasshouse Cassava (Manihot Esculenta Crantz). Plants (Basel) 6. 10.3390/plants6020022 PMC5489794 28555003

[B14] Burris K. P. Dlugosz E. M. Collins A. G. Stewart C. N. Lenaghan S. C. (2016). Development of a Rapid, Low-Cost Protoplast Transfection System for Switchgrass (Panicum Virgatum L.). Plant Cel. Rep. 35, 693–704. 10.1007/s00299-015-1913-7 PMC4757626 26685665

[B15] Cai X. Wei H. Liu C. Ren X. Thi L. T. Jeong B. R. (2020). Synergistic Effect of NaCl Pretreatment and PVP on Browning Suppression and Callus Induction from Petal Explants of Paeonia Lactiflora Pall. ‘Festival Maxima'. Plants (Basel) 9, 13. 10.3390/plants9030346 PMC7154888 32182923

[B16] Cai X. Kang X.-Y. (2014). Plant Regeneration from Cell Suspension-Derived Protoplasts of Populus × Beijingensis. *In Vitro* Cell.Dev.Biol.-Plant 50, 92–98. 10.1007/s11627-013-9540-x

[B17] Castelblanque L. García-Sogo B. Pineda B. Moreno V. (2010). Efficient Plant Regeneration from Protoplasts of Kalanchoe Blossfeldiana via Organogenesis. Plant Cel. Tiss. Organ. Cult. 100, 107–112. 10.1007/s11240-009-9617-8

[B18] Čermák T. Baltes N. J. Čegan R. Zhang Y. Voytas D. F. (2015). High-frequency, Precise Modification of the Tomato Genome. Genome Biol. 16, 232. 26541286 10.1186/s13059-015-0796-9 PMC4635538

[B19] Chamani E. Tahami S. k. (2016). Efficient Protocol for Protoplast Isolation and Plant Regeneration of Fritillaria Imperialis L. J. Agric. Sci. Technol. 18, 467–482.

[B20] Charrier A. Vergne E. Dousset N. Richer A. Petiteau A. Chevreau E. (2019). Efficient Targeted Mutagenesis in Apple and First Time Edition of Pear Using the CRISPR-Cas9 System. Front. Plant Sci. 10, 40. 10.3389/fpls.2019.00040 30787936 PMC6373458

[B21] Chen D. Shao Q. Yin L. Younis A. Zheng B. (2019). Polyamine Function in Plants: Metabolism, Regulation on Development, and Roles in Abiotic Stress Responses. Front. Plant Sci. 9, 1945. 10.3389/fpls.2018.01945 30687350 PMC6335389

[B22] Chen Z. Hsiao K.-C. Bornman C. H. (1994). Ploidy and Division Efficiency of Petiolar Protoplasts of Brassica Napus. Hereditas 120, 41–46.

[B23] Chikkala V. R. N. Nugent G. D. Dix P. J. Stevenson T. W. (2009). Regeneration from Leaf Explants and Protoplasts of *Brassica oleracea* Var. Botrytis (Cauliflower). Scientia Horticulturae 119, 330–334. 10.1016/j.scienta.2008.07.036

[B24] Craig W. Gargano D. Scotti N. Nguyen T. T. Lao N. T. Kavanagh T. A. (2005). Direct Gene Transfer in Potato: A Comparison of Particle Bombardment of Leaf Explants and PEG-Mediated Transformation of Protoplasts. Plant Cel. Rep. 24, 603–611. 10.1007/s00299-005-0018-0 16160836

[B112] Cui J. Kuligowska Mackenzie K. Eeckhaut T. Müller R. Lütken H. (2019). Protoplast Isolation and Culture from Kalanchoë Species: Optimization of Plant Growth Regulator Concentration for Efficient Callus Production. Plant Cel. Tiss. Organ. Cult. 138, 287–297. 10.1007/s11240-019-01624-4

[B25] Dai X.-M. Xiao W. Huang X. Zhao J.-T. Chen Y.-F. Huang X.-L. (2010). Plant Regeneration from Embryogenic Cell Suspensions and Protoplasts of Dessert Banana Cv. 'Da Jiao' (Musa Paradisiacal ABB Linn.) via Somatic Embryogenesis. *In Vitro* Cell.Dev.Biol.-Plant 46, 403–410. 10.1007/s11627-010-9314-7

[B26] Deryckere D. Eeckhaut T. Van Huylenbroeck J. Van Bockstaele E. (2012). Low Melting point Agarose Beads as a Standard Method for Plantlet Regeneration from Protoplasts within the Cichorium Genus. Plant Cel. Rep. 31, 2261–2269. 10.1007/s00299-012-1335-8 22926032

[B27] Dovzhenko A. Dal Bosco C. Meurer J. Koop H. U. (2003). Efficient Regeneration from Cotyledon Protoplasts in *Arabidopsis thaliana* . Protoplasma 222, 107–111. 10.1007/s00709-003-0011-9 14513316

[B28] Dovzhenko A. Koop H.-U. (2003). Sugarbeet ( Beta Vulgaris L.): Shoot Regeneration from Callus and Callus Protoplasts. Planta 217, 374–381. 10.1007/s00425-003-1006-7 14520564

[B113] Driver J. A. Kuniyuki A. H. (1984). In Vitro propagation of Paradox walnut Rootstock [Juglans Hindsii X Juglans Regia, Tissue Culture]. Hort. Sci. 19, 507–509.

[B29] Eeckhaut T. Van Houtven W. Bruznican S. Leus L. Van Huylenbroeck J. (2020). Somaclonal Variation in Chrysanthemum × Morifolium Protoplast Regenerants. Front. Plant Sci. 11, 607171. 10.3389/fpls.2020.607171 33391318 PMC7775395

[B30] Eeuwens C. J. (1976). Mineral Requirements for Growth and Callus Initiation of Tissue Explants Excised from Mature Coconut Palms (Cocos Nucifera) and Cultured *In Vitro* . Physiol. Plant 36, 23–28. 10.1111/j.1399-3054.1976.tb05022.x

[B31] Elmaghrabi A. Ochatt S. (2006). Isoenzymes and Flow Cytometry for the Assessment of True-To-Typeness of Calluses and Cell Suspensions of Barrel Medic Prior to Regeneration. Plant Cel. Tiss. Organ. Cult 85, 31–43. 10.1007/s11240-005-9046-2

[B114] Frearson E. M. Power J. B. Cocking E. C. (1973). The Isolation, Culture and Regeneration of Petunia Leaf Protoplasts. Developmental Biol. 33, 130–137. 10.1016/0012-1606(73)90169-3 4789596

[B32] Galun E. Raveh D. (1975). *In Vitro* culture of Tobacco Protoplasts: Survival of Haploid and Diploid Protoplasts Exposed to X-ray Radiation at Different Times after Isolation. Radiat. Bot. 15, 79–82. 10.1016/s0033-7560(75)80017-2

[B33] Gamborg O. L. Miller R. A. Ojima K. (1968). Nutrient Requirements of Suspension Cultures of Soybean Root Cells. Exp. Cel. Res. 50, 151–158. 10.1016/0014-4827(68)90403-5 5650857

[B34] Grzebelus E. Kruk M. Macko-Podgórni A. Grzebelus D. (2013). Response of Carrot Protoplasts and Protoplast-Derived Aggregates to Selection Using a Fungal Culture Filtrate of Alternaria Radicina. Plant Cel. Tiss. Organ. Cult. 115, 209–222. 10.1007/s11240-013-0353-8

[B35] Grzebelus E. Skop L. (2014). Effect of β-lactam Antibiotics on Plant Regeneration in Carrot Protoplast Cultures. *In Vitro* Cell.Dev.Biol.-Plant 50, 568–575. 10.1007/s11627-014-9626-0 25298730 PMC4182649

[B36] Grzebelus E. Szklarczyk M. Baranski R. (2012). An Improved Protocol for Plant Regeneration from Leaf- and Hypocotyl-Derived Protoplasts of Carrot. Plant Cel. Tiss. Organ. Cult. 109, 101–109. 10.1007/s11240-011-0078-5

[B115] Guan Q. Guo Y. Wei Y. Meng F. Zhang Z. (2010). Regeneration of Somatic Hybrids of Ginger via Chemical Protoplast Fusion. Plant Cel. Tiss. Organ. Cult. 102, 279–284. 10.1007/s11240-010-9730-8

[B37] Hu Q. Andersen S. B. Hansen L. N. (1999). Plant Regeneration Capacity of Mesophyll Protoplasts from Brassica Napus and Related Species. Plant Cel. Tissue Organ. Cult. 59, 189–196. 10.1023/a:1006417530587

[B116] Hu X. Yin Y. He T. (2015). Plant Regeneration from Protoplasts of Gentiana Macrophylla Pall. Using agar-pool Culture. Plant Cel. Tiss. Organ. Cult. 121, 345–351. 10.1007/s11240-014-0705-z

[B38] Hwang H.-H. Yu M. Lai E.-M. (2017). Agrobacterium-mediated Plant Transformation: Biology and Applications. Arab 15. 10.1199/tab.0186 PMC6501860 31068763

[B39] Imbrie-Milligan C. Kamo K. K. Hodges T. K. (1987). Microcallus Growth from maize Protoplasts. Planta 171, 58–64. 10.1007/bf00395067 24227270

[B40] Ishii S. (1988). Factors Influencing Protoplast Viability of Suspension-Cultured Rice Cells during Isolation Process. Plant Physiol. 88, 26–29. 10.1104/pp.88.1.26 16666273 PMC1055519

[B41] Jeong Y. Y. Lee H.-Y. Kim S. W. Noh Y.-S. Seo P. J. (2021). Optimization of Protoplast Regeneration in the Model Plant *Arabidopsis thaliana* . Plant Methods 17, 21. 10.1186/s13007-021-00720-x 33622383 PMC7901198

[B42] Jie E.-Y. Kim S.-W. Jang H.-R. In D.-S. Liu J.-R. (2011). Myo-inositol Increases the Plating Efficiency of Protoplast Derived from Cotyledon of Cabbage (*Brassica oleracea* Var. Capitata). J. Plant Biotechnol. 38, 69–76. 10.5010/jpb.2011.38.1.069

[B43] Jones A. M. P. Chattopadhyay A. Shukla M. Zoń J. Saxena P. K. (2012). Inhibition of Phenylpropanoid Biosynthesis Increases Cell wall Digestibility, Protoplast Isolation, and Facilitates Sustained Cell Division in American Elm (Ulmus Americana). BMC Plant Biol. 12, 75. 10.1186/1471-2229-12-75 22646730 PMC3464172

[B44] Jones A. M. P. Shukla M. R. Biswas G. C. G. Saxena P. K. (2015). Protoplast-to-plant Regeneration of American Elm (Ulmus Americana). Protoplasma 252, 925–931. 10.1007/s00709-014-0724-y 25359187

[B45] Kang H. H. Naing A. H. Kim C. K. (2020). Protoplast Isolation and Shoot Regeneration from Protoplast-Derived Callus of Petunia Hybrida Cv. Mirage Rose. Biology 9, 228. 10.3390/biology9080228 PMC7465674 32824283

[B46] Kao K. N. Michayluk M. R. (1975). Nutritional Requirements for Growth of Vicia Hajastana Cells and Protoplasts at a Very Low Population Density in Liquid media. Planta 126, 105–110. 10.1007/bf00380613 24430152

[B47] Karamian R. Ebrahimzadeh H. (2001). Plantlet Regeneration from Protoplast-Derived Embryogenic Calli of Crocus Cancellatus. Plant Cel. Tissue Organ. Cult. 65, 115–121. 10.1023/a:1010661620753

[B117] Karamian R. Ranjbar M. (2011). Somatic Embryogenesis and Plantlet Regeneration from Protoplast Culture of Muscari Neglectum Guss. Afr. J. Biotechnol. 10, 4602–4607.

[B48] Kästner U. Klocke E. Abel S. (2017). Regeneration of Protoplasts after Somatic Hybridisation of Hydrangea. Plant Cel. Tiss. Organ. Cult 129, 359–373. 10.1007/s11240-017-1183-x

[B49] Kaur N. D. Vyvadilová M. Klíma M. Bechyně M. (2006). A Simple Procedure for Mesophyll Protoplast Culture and Plant Regeneration in *Brassica oleracea* L. And Brassica Napus L. Czech. J. Genet. Plant Breed. 42, 103–110.

[B50] Kiełkowska A. Adamus A. (2012). An Alginate-Layer Technique for Culture of *Brassica oleracea* L. protoplastsIn Vitro. Cel. Dev. Biol. Plant. 48, 265–273. 10.1007/s11627-012-9431-6 PMC3337407 22593638

[B51] Kiełkowska A. Adamus A. (2021). Exogenously Applied Polyamines Reduce Reactive Oxygen Species, Enhancing Cell Division and the Shoot Regeneration from *Brassica oleracea* L. Var. Capitata Protoplasts. Agronomy 11, 735.

[B52] Kiełkowska A. Adamus A. (2019). Peptide Growth Factor Phytosulfokine-α Stimulates Cell Divisions and Enhances Regeneration from *B. oleracea* Var. Capitata L. Protoplast Culture. J. Plant Growth Regul. 38, 931–944.

[B53] Kiełkowska A. Grzebelus E. Lis-Krzyścin A. Maćkowska K. (2019). Application of the Salt Stress to the Protoplast Cultures of the Carrot (Daucus Carota L.) and Evaluation of the Response of Regenerants to Soil Salinity. Plant Cell Tissue Organ Cult. PCTOC 137, 379–395.

[B54] Klejdus B. Kováčik J. Babula P. (2013). PAL Inhibitor Evokes Different Responses in Two Hypericum Species. Plant Physiol. Biochem. 63, 82–88. 10.1016/j.plaphy.2012.11.019 23254282

[B55] Klimek-Chodacka M. Kadluczka D. Lukasiewicz A. Malec-Pala A. Baranski R. Grzebelus E. (2020). Effective Callus Induction and Plant Regeneration in Callus and Protoplast Cultures of Nigella Damascena L. Plant Cel. Tiss. Organ. Cult 143, 693–707. 10.1007/s11240-020-01953-9

[B56] Kobayashi S. Kameya T. Ichihashi S. (1993). Plant Regeneration from Protoplasts Derived from Callus of Phalaenopsis. Plant Tissue Cult. Lett. 10, 267–270. 10.5511/plantbiotechnology1984.10.267

[B57] Kyozuka J. Hayashi Y. Shimamoto K. (1987). High Frequency Plant Regeneration from rice Protoplasts by Novel Nurse Culture Methods. Mol. Gen. Genet. 206, 408–413. 10.1007/bf00428879

[B58] Lassoued R. Phillips P. W. B. Macall D. M. Hesseln H. Smyth S. J. (2021). Expert Opinions on the Regulation of Plant Genome Editing. Plant Biotechnol. J. 10.1111/pbi.13597 PMC8196660 33834596

[B59] Lee M. H. Lee J. Choi S. A. Kim Y.-S. Koo O. Choi S. H. (2020). Efficient Genome Editing Using CRISPR-Cas9 RNP Delivery into Cabbage Protoplasts via Electro-Transfection. Plant Biotechnol. Rep. 14, 695–702. 10.1007/s11816-020-00645-2

[B60] Liang Z. Chen K. Zhang Y. Liu J. Yin K. Qiu J.-L. (2018). Genome Editing of Bread Wheat Using Biolistic Delivery of CRISPR/Cas9 *In Vitro* Transcripts or Ribonucleoproteins. Nat. Protoc. 13, 413–430. 10.1038/nprot.2017.145 29388938

[B61] Lin C.-S. Hsu C.-T. Yang L.-H. Lee L.-Y. Fu J.-Y. Cheng Q.-W. (2018). Application of Protoplast Technology to CRISPR/Cas9 Mutagenesis: from Single-Cell Mutation Detection to Mutant Plant Regeneration. Plant Biotechnol. J. 16, 1295–1310. 10.1111/pbi.12870 29230929 PMC5999315

[B62] Lin Q. Zong Y. Xue C. Wang S. Jin S. Zhu Z. (2020). Prime Genome Editing in rice and Wheat. Nat. Biotechnol. 38, 582–585. 10.1038/s41587-020-0455-x 32393904

[B63] Lopez-Arellano M. Dhir S. Albino C. N. Santiago A. Morris T. Dhir S. K. (2014). Somatic Embryogenesis and Plantlet Regeneration from Protoplast Culture of Stevia rebaudiana. Br. Biotechnol. J. 5, 1–12.

[B64] Lukaszewska E. Virden R. Sliwinska E. (2012). Hormonal Control of Endoreduplication in Sugar Beet (Beta Vulgaris L.) Seedlings Growing *In Vitro* . Plant Biol. (Stuttg) 14, 216–222. 10.1111/j.1438-8677.2011.00477.x 21973015

[B65] Ma X. Zhang X. Liu H. Li Z. (2020). Highly Efficient DNA-free Plant Genome Editing Using Virally Delivered CRISPR-Cas9. Nat. Plants 6, 773–779. 10.1038/s41477-020-0704-5 32601419

[B66] Maćkowska K. Jarosz A. Grzebelus E. (2014). Plant Regeneration from Leaf-Derived Protoplasts within the Daucus Genus: Effect of Different Conditions in Alginate Embedding and Phytosulfokine Application. Plant Cell Tissue Organ Cult. PCTOC 117, 241–252.

[B67] Magnien E. Dalschaert X. Faraoni-Sciamanna P. (1982). Transmission of a Cytological Heterogeneity from the Leaf to the Protoplasts in Culture. Plant Sci. Lett. 25, 291–303. 10.1016/0304-4211(82)90159-6

[B68] Majewska-Sawka A. Niklas A. Jażdżewska E. (1997). The Effect of Polyamines on the Development of Sugar Beet Protoplasts. Biol. Plant. 39, 561–567. 10.1023/a:1000926714622

[B69] Masani M. Y. A. Noll G. Parveez G. K. A. Sambanthamurthi R. Prüfer D. (2013). Regeneration of Viable Oil palm Plants from Protoplasts by Optimizing media Components, Growth Regulators and Cultivation Procedures. Plant Sci. 210, 118–127. 10.1016/j.plantsci.2013.05.021 23849119

[B70] Masson J. Paszkowski J. (1992). The Culture Response ofArabidopsis Thalianaprotoplasts Is Determined by the Growth Conditions of Donor Plants. Plant J. 2, 829–833. 10.1111/j.1365-313x.1992.tb00153.x

[B71] Meyer L. Serek M. Winkelmann T. (2009). Protoplast Isolation and Plant Regeneration of Different Genotypes of Petunia and Calibrachoa. Plant Cel. Tiss. Organ. Cult. 99, 27–34. 10.1007/s11240-009-9572-4

[B72] Mizuhiro M. Kenichi Y. Ito K. Kadowaki S. Ohashi H. Mii M. (2001). Plant Regeneration from Cell Suspension-Derived Protoplasts of Primula Malacoides and Primula Obconica. Plant Sci. 160, 1221–1228. 10.1016/s0168-9452(01)00390-9 11337079

[B73] Murashige T. Skoog F. (1962). A Revised Medium for Rapid Growth and Bio Assays with Tobacco Tissue Cultures. Physiol. Plant 15, 473–497. 10.1111/j.1399-3054.1962.tb08052.x

[B74] Nagata T. Ishii S. (1979). A Rapid Method for Isolation of Mesophyll Protoplasts. Can. J. Bot. 57, 4. 10.1139/b79-226

[B75] Nassour M. Dorion N. (2002). Plant Regeneration from Protoplasts of Micropropagated Pelargonium X Hortorum ‘Alain': Effect of Some Environmental and Medium Factors on Protoplast System Efficiency. Plant Sci. 163, 169–176. 10.1016/s0168-9452(02)00093-6

[B118] Nirmal Babu K. Samsudeen K. Divakaran M. Pillai G. S. Sumathi V. Praveen K. (2016). “Protocols for In Vitro Propagation, Conservation, Synthetic Seed Production, Embryo Rescue, Microrhizome Production, Molecular Profiling, and Genetic Transformation in Ginger (Zingiber Officinale Roscoe.),” in Protocols for in Vitro Cultures and Secondary Metabolite Analysis of Aromatic and Medicinal Plants. Methods in Molecular Biology. Editor Jain S. M. Second Edition (New York, NY: Springer), 403–426. 10.1007/978-1-4939-3332-7_28 27108333

[B76] Nitsch J. P. Nitsch C. (1969). Haploid Plants from Pollen Grains. Science 163, 85–87. 10.1126/science.163.3862.85 17780179

[B77] Nybakken L. Keski-Saari S. Falck M. A. Julkunen-Tiitto R. (2007). Restoration of Secondary Metabolism in Birch Seedlings Relieved from PAL-Inhibitor. Trees 21, 273–281. 10.1007/s00468-006-0120-0

[B78] Ochatt S. J. Mousset-Déclas C. Rancillac M. (2000). Fertile Pea Plants Regenerate from Protoplasts when Calluses Have Not Undergone Endoreduplication. Plant Sci. 156, 177–183. 10.1016/s0168-9452(00)00250-8 10936524

[B79] Osakabe Y. Liang Z. Ren C. Nishitani C. Osakabe K. Wada M. (2018). CRISPR-Cas9-mediated Genome Editing in Apple and grapevine. Nat. Protoc. 13, 2844–2863. 10.1038/s41596-018-0067-9 30390050

[B119] Park J. Choi S. Park S. Yoon J. Park A. Y. Choe S. (2019). “DNA-free Genome Editing via Ribonucleoprotein (RNP) Delivery of CRISPR/Cas in Lettuce,” in Plant Genome Editing with CRISPR Systems: Methods and Protocols. Methods in Molecular Biology. Editor Qi Y. (New York, NY: Springer), 337–354. 10.1007/978-1-4939-8991-1_25 30610648

[B80] Pauk J. Fekete S. Vilkki J. Pulli S. (1991). Protoplast Culture and Plant Regeneration of Different Agronomically Important Brassica Species and Varieties. AFSci 63, 371–378. 10.23986/afsci.72416

[B81] Pearce R. S. Cocking E. C. (1973). Behaviour in Culture of Isolated Protoplasts from .Paul's Scarlet. Rose Suspension Culture Cells. Protoplasma 77, 165–180. 10.1007/bf01276755

[B82] Prakash A. H. Rao K. S. Kumar M. U. (1997). Plant Regeneration from Protoplasts ofCapsicum Annuum L. Cv. California Wonder. J. Biosci. 22, 339–344. 10.1007/bf02703236

[B83] Prange A. N. S. Bartsch M. Serek M. Winkelmann T. (2010a). Regeneration of Different Cyclamen Species via Somatic Embryogenesis from Callus, Suspension Cultures and Protoplasts. Scientia Horticulturae 125, 442–450. 10.1016/j.scienta.2010.04.018

[B84] Prange A. N. S. Serek M. Bartsch M. Winkelmann T. (2010b). Efficient and Stable Regeneration from Protoplasts of Cyclamen Coum Miller via Somatic Embryogenesis. Plant Cel. Tiss. Organ. Cult. 101, 171–182. 10.1007/s11240-010-9674-z

[B85] Qin Y. H. Teixeira da Silva J. A. Bi J. H. Zhang S. L. Hu G. B. (2011). Response of *In Vitro* Strawberry to Antibiotics. Plant Growth Regul. 65, 183–193. 10.1007/s10725-011-9587-9

[B120] Rahmani M.-S. Pijut P. M. Shabanian N. (2016). Protoplast Isolation and Genetically True-to-type Plant Regeneration from Leaf- and Callus-Derived Protoplasts of Albizia Julibrissin. Plant Cel. Tiss. Organ. Cult. 127, 475–488. 10.1007/s11240-016-1072-8

[B86] Reustle G. Natter I. (1994). Effect of Polyvinylpyrrolidone and Activated Charcoal on Formation of Microcallus from grapevine Protoplasts (Vitis sp.). VITIS - J. Grapevine Res. 33, 117–121.

[B87] Rezazadeh R. Niedz R. P. (2015). Protoplast Isolation and Plant Regeneration of Guava (Psidium Guajava L.) Using Experiments in Mixture-Amount Design. Plant Cel. Tiss. Organ. Cult. 122, 585–604. 10.1007/s11240-015-0790-7

[B88] Ruesink A. W. (1978). Leucine Uptake and Incorporation by Convolvulus Tissue Culture Cells and Protoplasts under Severe Osmotic Stress. Physiol. Plant 44, 48–56. 10.1111/j.1399-3054.1978.tb01612.x

[B121] Sahab S. Hayden M. J. Mason J. Spangenberg G. (2019). “Mesophyll Protoplasts and PEG-Mediated Transfections: Transient Assays and Generation of Stable Transgenic Canola Plants,” in Transgenic Plants: Methods and Protocols. Methods in Molecular Biology. Editors Kumar S. Barone P. Smith M. (New York, NY: Springer), 131–152. 10.1007/978-1-4939-8778-8_10 30415334

[B89] Sakamoto Y. Kawamura A. Suzuki T. Segami S. Maeshima M. Polyn S. (2021). Transcriptional Activation of Auxin Biosynthesis Drives Developmental Reprogramming of Differentiated Cells. bioRxiv. 10.1101/2021.06.26.450054 PMC9614439 35922895

[B90] Sauer N. J. Narváez-Vásquez J. Mozoruk J. Miller R. B. Warburg Z. J. Woodward M. J. (2016). Oligonucleotide-Mediated Genome Editing Provides Precision and Function to Engineered Nucleases and Antibiotics in Plants. Plant Physiol. 170, 1917–1928. 10.1104/pp.15.01696 26864017 PMC4825113

[B91] Saxena P. K. Gill R. (1986). Removal of browning and Growth Enhancement by Polyvinylpolypyrrolidone in Protoplast Cultures of Cyamopsis Tetragonoloba L. Biol. Plant 28, 313–315. 10.1007/bf02902302

[B92] Shahin E. A. (1985). Totipotency of Tomato Protoplasts. Theoret. Appl. Genet. 69, 235–240. 10.1007/bf00662431 24253814

[B93] Shan Q. Wang Y. Li J. Zhang Y. Chen K. Liang Z. (2013). Targeted Genome Modification of Crop Plants Using a CRISPR-Cas System. Nat. Biotechnol. 31, 686–688. 10.1038/nbt.2650 23929338

[B94] Sheng X. Zhao Z. Yu H. Wang J. Xiaohui Z. Gu H. (2011). Protoplast Isolation and Plant Regeneration of Different Doubled Haploid Lines of Cauliflower (*Brassica oleracea* Var. Botrytis). Plant Cel. Tiss. Organ. Cult. 107, 513–520. 10.1007/s11240-011-0002-z

[B95] Shulse C. N. Cole B. J. Ciobanu D. Lin J. Yoshinaga Y. Gouran M. (2019). High-Throughput Single-Cell Transcriptome Profiling of Plant Cell Types. Cel. Rep. 27, 2241–2247. 10.1016/j.celrep.2019.04.054 PMC6758921 31091459

[B122] Staba E. J. (1969). Plant Tissue Culture as a Technique for the Phytochemist. Recent Adv. Phytochem. 2, 75–105.

[B96] Subburaj S. Chung S. J. Lee C. Ryu S.-M. Kim D. H. Kim J.-S. (2016). Site-directed Mutagenesis in Petunia × Hybrida Protoplast System Using Direct Delivery of Purified Recombinant Cas9 Ribonucleoproteins. Plant Cel. Rep. 35, 1535–1544. 10.1007/s00299-016-1937-7 26825596

[B97] Svitashev S. Schwartz C. Lenderts B. Young J. K. Mark Cigan A. (2016). Genome Editing in maize Directed by CRISPR-Cas9 Ribonucleoprotein Complexes. Nat. Commun. 7, 13274. 10.1038/ncomms13274 27848933 PMC5116081

[B123] Tahami S. K. Chamani E. Zare N. (2014). Plant Regeneration from Protoplasts of Lilium Ledebourii (Baker) Boiss. J. Agric. Sci. Technology 16, 1133–1144.

[B98] Tang W. Harris L. C. Outhavong V. Newton R. J. (2004). Antioxidants Enhance *In Vitro* Plant Regeneration by Inhibiting the Accumulation of Peroxidase in Virginia pine (Pinus Virginiana Mill.). Plant Cel. Rep. 22, 871–877. 10.1007/s00299-004-0781-3 15042408

[B99] Tomiczak K. Mikuła A. Sliwinska E. Rybczyński J. J. (2015). Autotetraploid Plant Regeneration by Indirect Somatic Embryogenesis from Leaf Mesophyll Protoplasts of Diploid Gentiana Decumbens L.F. *In Vitro* Cell.Dev.Biol.-Plant 51, 350–359. 10.1007/s11627-015-9674-0 26097374 PMC4471314

[B100] Vessabutr S. Grant W. F. (1995). Isolation, Culture and Regeneration of Protoplasts from Birdsfoot Trefoil (*Lotus corniculatus*). Plant Cel. Tiss. Organ. Cult. 41, 9–15. 10.1007/bf00124081

[B101] Wójcik A. Rybczyński J. J. (2015). Electroporation and Morphogenic Potential of Gentiana Kurroo (Royle) Embryogenic Cell Suspension Protoplasts. bta 1, 19–29. 10.5114/bta.2015.54170

[B102] Woo J. W. Kim J. Kwon S. I. Corvalán C. Cho S. W. Kim H. (2015). DNA-free Genome Editing in Plants with Preassembled CRISPR-Cas9 Ribonucleoproteins. Nat. Biotechnol. 33, 1162–1164. 10.1038/nbt.3389 26479191

[B103] Wu F.-H. Shen S.-C. Lee L.-Y. Lee S.-H. Chan M.-T. Lin C.-S. (2009). Tape-Arabidopsis Sandwich - a Simpler Arabidopsis Protoplast Isolation Method. Plant Methods 5, 16. 10.1186/1746-4811-5-16 19930690 PMC2794253

[B104] Xiao L. Zhang L. Yang G. Zhu H. He Y. (2012). Transcriptome of Protoplasts Reprogrammed into Stem Cells in Physcomitrella Patens. PLoS ONE 7, e35961. 10.1371/journal.pone.0035961 22545152 PMC3335808

[B105] Yang H. Matsubayashi Y. Nakamura K. Sakagami Y. (1999). Oryza Sativa PSK Gene Encodes a Precursor of Phytosulfokine-Alpha, a Sulfated Peptide Growth Factor Found in Plants. Proc. Natl. Acad. Sci. 96, 13560–13565. 10.1073/pnas.96.23.13560 10557360 PMC23987

[B106] Yong J. W. Ge L. Ng Y. F. Tan S. N. (2009). The Chemical Composition and Biological Properties of Coconut (Cocos Nucifera L.) Water. Molecules 14, 5144–5164. 10.3390/molecules14125144 20032881 PMC6255029

[B107] Yu J. Tu L. Subburaj S. Bae S. Lee G.-J. (2020). Simultaneous Targeting of Duplicated Genes in Petunia Protoplasts for Flower Color Modification via CRISPR-Cas9 Ribonucleoproteins. Plant Cel. Rep. 10.1007/s00299-020-02593-1 32959126

[B151] Zafar Y. Nenz E. Damiani F. Pupilli F. Arcioni S. (1995). Plant regeneration from explant and protoplast derived calluses of Medicago littoralis. Plant Cell Tissue Organ Cult. 41: 41–48.

[B108] Zhang Y. Liang Z. Zong Y. Wang Y. Liu J. Chen K. (2016). Efficient and Transgene-free Genome Editing in Wheat through Transient Expression of CRISPR/Cas9 DNA or RNA. Nat. Commun. 7, 12617. 10.1038/ncomms12617 27558837 PMC5007326

[B109] Zhang Y. Malzahn A. A. Sretenovic S. Qi Y. (2019). The Emerging and Uncultivated Potential of CRISPR Technology in Plant Science. Nat. Plants 5, 778–794. 10.1038/s41477-019-0461-5 31308503

[B110] Zhou J. Wang B. Zhu L. (2005). Conditioned Culture for Protoplasts Isolated from chrysanthemum: An Efficient Approach. Colloids Surf. B: Biointerfaces 45, 113–119. 10.1016/j.colsurfb.2005.07.012 16154327

